# Monitoring the Structural Changes in Iridium Nanoparticles during Oxygen Evolution Electrocatalysis with *Operando* X-ray Total Scattering

**DOI:** 10.1021/jacs.4c08149

**Published:** 2024-09-30

**Authors:** Rebecca K. Pittkowski, Stefanie Punke, Andy S. Anker, Aline Bornet, Nicolas Pierre Louis Magnard, Nicolas Schlegel, Laura G. Graversen, Jonathan Quinson, Alexandra Dworzak, Mehtap Oezaslan, Jacob J. K. Kirkensgaard, Marta Mirolo, Jakub Drnec, Matthias Arenz, Kirsten M. Ø. Jensen

**Affiliations:** † Department of Chemistry, University of Copenhagen, Universitetsparken 5, 2100 Copenhagen, Denmark; ‡ Department of Chemistry, Biochemistry and Pharmaceutical Sciences, University of Bern, Freiestrasse 3, 3012 Bern, Switzerland; § Biological and Chemical Engineering Department, Aarhus University, 40 Åbogade, 8200 Aarhus, Denmark; ∥ Technical Electrocatalysis Laboratory, Institute of Technical Chemistry, Technische Universität Braunschweig, 38106 Braunschweig, Germany; ⊥ Niels Bohr Institute, University of Copenhagen, Universitetsparken 5, 2100 Copenhagen, Denmark; # Department of Food Science, University of Copenhagen, Rolighedsvej 26, 1958 Frederiksberg, Denmark; ∇ ESRF—The European Synchrotron, 71 Avenue des Martyrs, Grenoble 38000, France

## Abstract

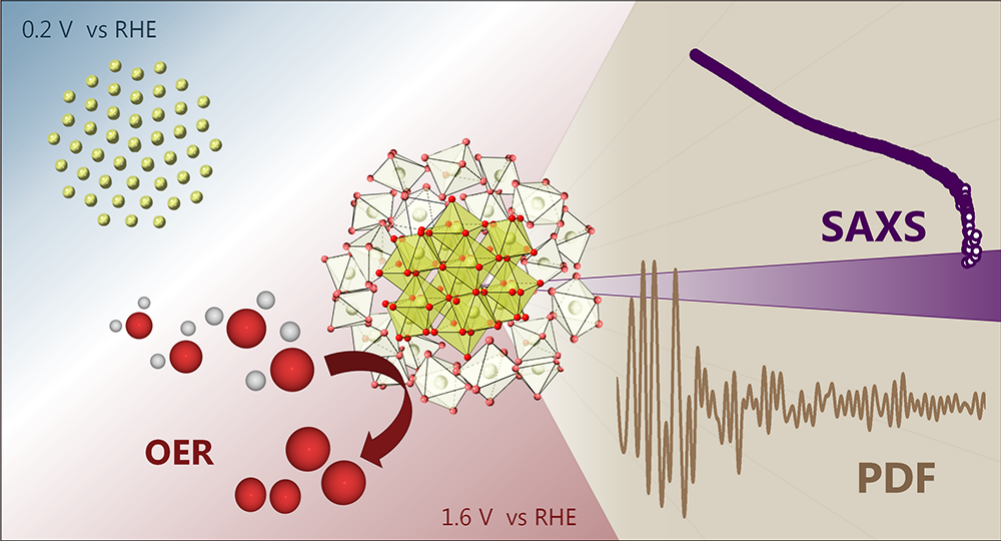

Understanding the structure of nanoparticles under (electro)catalytic operating conditions is crucial for uncovering structure–property relationships. By combining *operando* X-ray total scattering and pair distribution function analysis with *operando* small-angle X-ray scattering (SAXS), we obtained comprehensive structural information on ultrasmall (<3 nm) iridium nanoparticles and tracked their changes during oxygen evolution reaction (OER) in acid. When subjected to electrochemical conditions at reducing potentials, the metallic Ir nanoparticles are found to be decahedral. The iridium oxide formed in the electrochemical oxidation contains small rutile-like clusters composed of edge- and corner-connected [IrO_6_] octahedra of a very confined range. These rutile domains are smaller than 1 nm. Combined with complementary SAXS data analysis to extract the particle size, we find that the OER-active iridium oxide phase lacks crystalline order. Additionally, we observe an iridium oxide contraction under OER conditions, which is confirmed by *operando* X-ray absorption spectroscopy. Our results highlight the need for multitechnique *operando* studies for a complete understanding of the electrochemically formed Ir oxide active in OER.

## Introduction

Water electrolysis for hydrogen production is a key technology in the storage of intermittent renewable electricity.^1^ Proton exchange membrane (PEM) electrolyzers offer the highest potential for large-scale hydrogen production due to their high efficiency and operational safety, functioning at high pressures and current densities.^1,2^ Currently, iridium-based electrocatalysts remain the only viable anode catalysts for PEM electrolyzers, providing excellent stability and activity in the acidic oxygen evolution reaction (OER).^3−5^ However, iridium is a very limited resource as one of the scarcest elements on Earth.^6^ Therefore, it is crucial to significantly reduce the catalyst loading (in g_Ir_ kW^–1^) to enable large-scale implementation of PEM electrolyzers using iridium catalysts.^6^ To fully exploit the iridium used as a catalyst, a high dispersion (surface-to-mass ratio) is essential, and ultrasmall iridium nanoparticles spread on a high surface area support material provide such a catalyst with high dispersion.^7^ So far, it has been established that electrochemically formed iridium oxides that are poorly crystalline/amorphous outperform crystalline rutile oxides of Ir.^8−11^ Moreover, the crystal structure and oxidation state^12^ of the iridium oxide plays an important role in catalyst activity and stability.^13,14^ However, determining the atomic structure of the electrochemically formed iridium oxide from ultrasmall iridium nanoparticles in the reaction environment remains challenging, meaning that structure/property relations are still not well understood.

It is widely acknowledged that the structure of most synthesized materials should be considered as precatalysts rather than the actual catalyst phase, which only forms in the electrochemical environment under reaction conditions.^15−17^ In oxygen evolution catalysis, for example, the oxidative potential that is required to drive the conversion of water to oxygen often also leads to redox processes in the catalyst material. Therefore, merely characterizing the as-synthesized catalysts is rarely sufficient to understand the atomic structure of the catalysts under reaction conditions.^18,19^ To conduct any theoretical, mechanistic studies of the oxygen evolution reaction on a specific catalyst material, it is vital to identify the structural motif forming the catalytically active site.^8,13^ As a consequence, numerous *operando* studies of iridium-based electrocatalysts have been conducted to elucidate the catalyst structure under operating conditions. These studies have mostly been focused on different spectroscopic techniques^20^ including Raman spectroscopy,^21−25^ X-ray photoelectron spectroscopy (XPS),^26−29^ and X-ray absorption spectroscopy (XAS).^3,21,30,31^ These studies have shown that the oxidation state of iridium is crucial for OER activity. Changes in oxidation state need to be accompanied by changes in the atomic structure. For ultrasmall nanoparticles, it is however difficult to study these changes. While XRD is the most suitable technique to study transformations of crystalline materials in electrochemical environments,^16,32^ it does not provide information for ultrasmall iridium nanoparticles. Raman and XPS do not provide structural information and XAS analysis is restricted to the first coordination shell. We here apply *operando* X-ray total scattering with pair distribution function (PDF) analysis combined with small-angle X-ray scattering (SAXS) to unravel the structural changes of iridium in the electrochemical environment.

With SAXS it is possible to determine the particle size and morphology of catalyst particles, for example, showing particle agglomeration and atomic rearrangements as previously reported in in situ and *operando* SAXS studies of platinum fuel cell catalysts^33−36^ and iridium OER catalysts^3^ in electrochemical cells. While SAXS gives information on the nanoscale morphology, information on atomic structure is, nevertheless, still missing. Here, PDF analysis can be applied. PDF is now a well-established technique for investigating the atomic structure of amorphous,^37−39^ disordered,^40−42^ and nanostructured materials.^42−46^ Compared to conventional XRD, where only Bragg peaks are considered in the data analysis, total scattering techniques take diffuse scattering into account, which makes it possible to extract structural information on materials with only local structural order. The PDF is the Fourier transform of total scattering data and represents a histogram of interatomic distances in the sample. It is thus an intuitive method for the analysis of scattering data. PDF analysis has been used to study catalyst materials under reaction conditions in e.g. heterogeneous thermal catalysis^47−50^ as well as electrochemical transformations of battery materials.^51−53^ In the field of electrocatalysis, however, research using X-ray total scattering combined with PDF analysis of catalyst materials under reaction conditions remains scarce: a PEM fuel cell optimized for X-ray total scattering has been developed and used to study alloyed nanoparticles as fuel cell catalysts.^54−56^ Despite its relevance for studies of nanocrystalline materials such as Ir nanoparticles, to the best of our knowledge, there have been no reported X-ray total scattering *operando* studies conducted on ultrasmall nanoparticles functioning as electrocatalysts in liquid electrolyte electrochemical cells.

In this work, we investigate the structural changes of ultrasmall Ir nanoparticles in an electrochemical environment using X-ray total scattering with PDF analysis combined with SAXS, complemented by *operando* XAS. We investigate the atomic structure of the electrochemically formed iridium oxide in acid media under applied oxidative potentials and track the structural modifications during oxygen evolution electrocatalysis. Our results show that the nanoparticle oxidation results in rutile-structured iridium oxides of <1 nm domain size with no significant increase in particle size during oxidation. An iridium oxide exhibiting only local structure is identified as the OER active phase, highlighting the need for structural studies of electrocatalysts under operating conditions.

## Results and Discussion

### Electrochemical Protocol and Operando PDF Overview

Ultrasmall iridium nanoparticles were prepared by a colloidal surfactant-free synthesis approach^7,57,58^ and immobilized on a high surface area carbon support.^7^ Carbon was selected as the support material to maintain dispersion while minimizing any particle–support interactions.^7,59^ Electrodes were prepared by vacuum filtration of the Ir/C catalyst powder (50 wt %, 200 μg_Ir_/cm^2^) onto a gas diffusion layer (GDL) to obtain ca. 10 μm-thick catalyst layers. All *operando* scattering experiments were performed in a three-electrode electrochemical cell^60^ that allows for collecting X-ray scattering data during the electrochemical experiments. Details on the catalyst, electrode preparation, and *operando* experiments are given in Section 1—Materials and Methods of the Supporting Information (SI, Figures S1–S3). The average nanoparticle size was determined as 1.7 ± 0.3 nm by statistical analysis of HR-TEM images (Supporting Information, Figure S4). After immobilization, no significant changes in particle size and morphology were observed, and the ultrasmall Ir nanoparticles appear well dispersed on the support material (Figure S5). Cyclic voltammograms (Figure S6) were recorded of the prepared Ir/C GDL electrode to confirm that the catalytic performance of the prepared catalyst for OER agrees with literature reports.^7,61^

**Figure 1:**
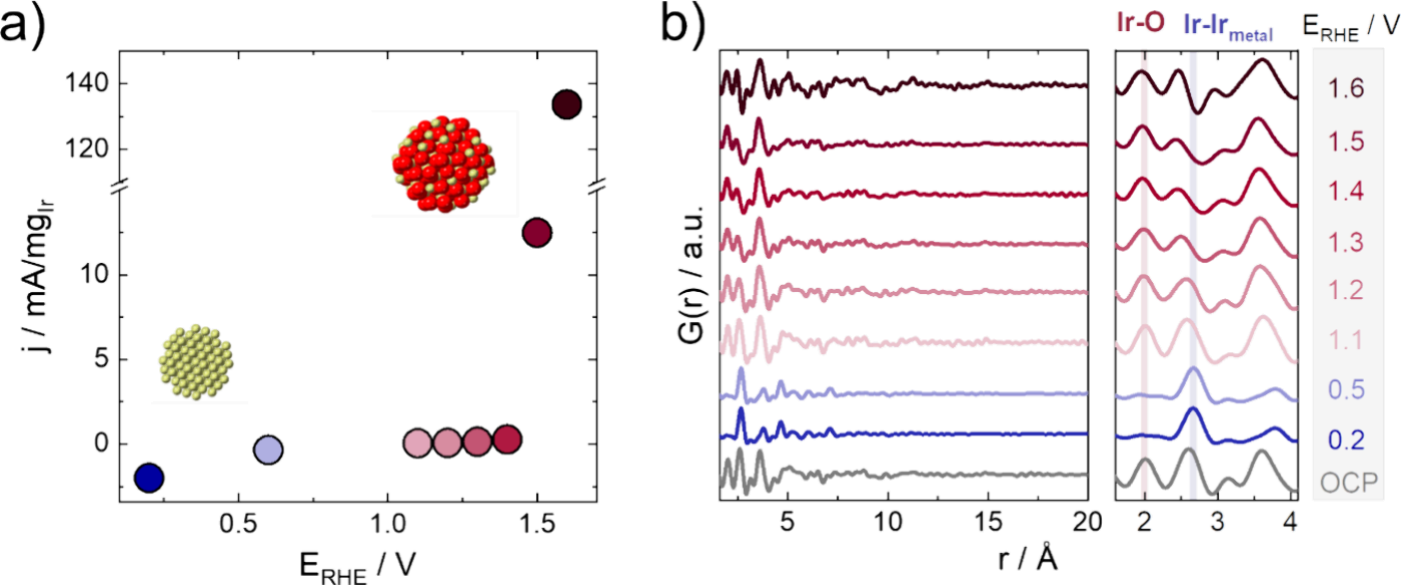
(a) Ir-based currents collected during the potential holds (chronoamperometry) of 10 min. The currents were averaged over the last 100 s, and (b) corresponding *operando* PDFs extracted from X-ray total scattering data collected at the respective potentials, with zoom-in on the local range of the PDFs in the region 2–4 Å. The Ir–O distance of the oxide is highlighted in light red and the Ir–Ir metal pair distance in light blue, respectively.

X-ray total scattering data were collected at a row of subsequentially applied electrochemical potential steps as shown in Figure 1. To have a defined reference point of the precatalyst structure, we first electrochemically reduced any surface oxide that had formed due to exposure to air.^59^ Starting from the open circuit potential (OCP) at ca. 0.8 V vs RHE, we applied a low reduction potential (0.2 V vs RHE). Subsequently, the obtained metallic iridium nanoparticles are oxidized at increasingly positive potentials. We applied steps of more and more oxidative potentials up to a point where a significant OER current was measured. In this way, we proceeded from metallic iridium to iridium oxidation and into the oxygen evolution regime. The mass activity derived from the chronoamperometric measurements is shown as a function of applied potential in Figure 1a. By collecting X-ray total scattering data during the electrochemical protocol combined with pair distribution function (PDF) analysis, we can follow the changes in the atomic structure of the nanoparticles at the respective applied potentials. The overview in Figure 1b shows the PDFs extracted at each potential step. Peaks in the PDF represent interatomic distances in the material. In all stages of the experiment, PDF peaks are only visible up to around 10 Å, which agrees with the nanosize nature of the prepared nanoparticles.^7,57^

The PDF peak at ca. 2 Å, highlighted in light red in the right panel in Figure 1b, corresponds to the first Ir–O distance. By analyzing the intensity of this peak, we can follow the oxide content in the nanoparticle structure. At OCP, the particles are partially oxidized and the Ir–O peak is visible, as seen from the PDF plotted in gray in Figure 1b. Once a low potential is applied (0.2 V vs RHE), a cathodic current is recorded (see Figure 1a and SI, Figure S7), indicating the electrochemical reduction of the IrO_*x*_ surface nanoparticles. The characteristic distances in the PDF of the metallic nanoparticles (blue PDFs in Figure 1b) differ significantly from the air-oxidized nanoparticles, e.g., in the absence of an Ir–O peak. Instead, a strong Ir–Ir peak (2.7 Å) from the metallic nanoparticles dominates the PDF, which is highlighted in light blue in Figure 1b.

We followed the oxidation of the iridium nanoparticles by gradually increasing the potential in the anodic direction. At 0.5 V vs RHE, the iridium remains metallic. Once we apply oxidative potentials, from 1.1 V vs RHE, increasing anodic currents are recorded for each potential step, corresponding to iridium oxidation as seen in Figure 1a. At this point, the Ir–O peak reappears in the PDFs, showing the oxide character of the catalyst at these applied potentials. At 1.5 and 1.6 V vs RHE, significant OER currents are recorded, as shown in Figure 1a. The corresponding Tafel plots (*E* vs log *j*) of the *operando* experiment are included in the SI (Figure S8), displaying a distinction between the Ir oxidation and the OER regime. The PDFs collected at these potentials thus allow us to extract information on the atomic structure of the active catalyst during the OER.

To ensure the reproducibility of both the electrochemical OER performance as well as the structural information obtained, the experiment was repeated for a second catalyst film. The same behavior in the reduction and oxidation of the Ir nanoparticles can be followed in the PDFs of the repeat measurement as shown in the Supporting Information, Figures S9–S10. Note that we observed a small contribution from a minor crystalline phase for sample 1 at 1.6 V vs RHE, which we attributed to an incomplete subtraction of the scattering arising from polyetheretherketone (PEEK) material of the *operando* cell (see SI, Figures S11 and S12). This may be due to a loss of catalyst material at high current densities (>130 mA/cm^2^) during strong bubble formation, which leads to a relatively more intense background signal. Importantly, the main processes are similar for the two samples. In agreement with previous results,^57^ we find the electrochemical oxidation process to be irreversible (Figure S13). To address the experimental difficulties of performing *operando* PDF experiments, we have included a discussion of the challenges related to the experiments in the methods section of the Supporting Information.

### Structure of Electrochemically Reduced Ir Nanoparticles

**Figure 2:**
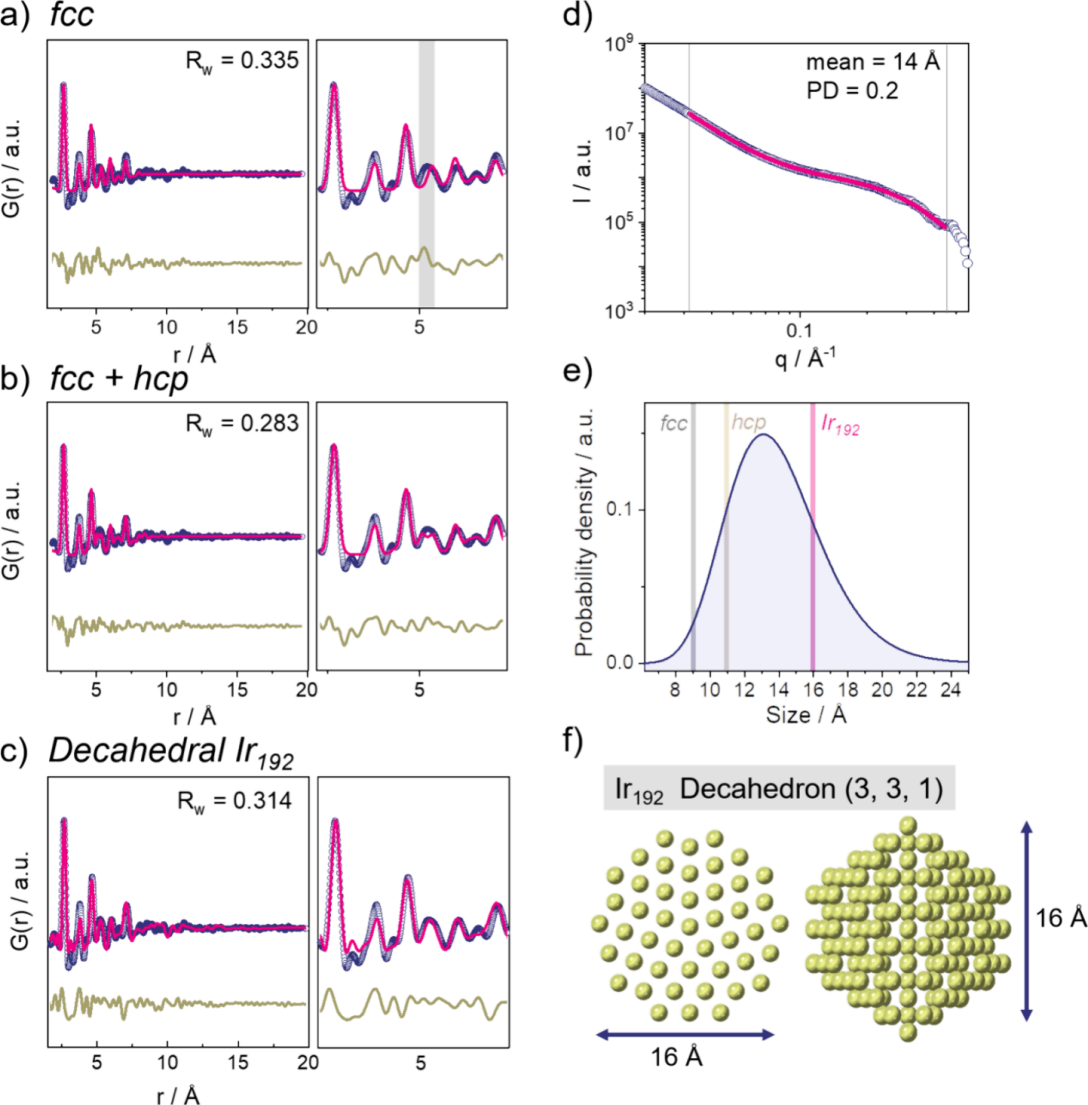
Characterization of metallic nanoparticles obtained through electrochemical reduction (potential hold at 0.2 V vs RHE) with (a) fit of an *fcc* phase model, (b) two-phase model with both *fcc* and *hcp* phase to the PDF data, and (c) best fit of a model of decahedral particles to the PDF: the decahedral particle is constructed with three layers parallel to the 5-fold axis, three layers truncated perpendicular to the pentagonal edges and one layer truncated perpendicular to the five apical vertices (192 Ir atoms). Figure (d) shows the fit to the SAXS data of a model of polydisperse spherical particles, as well as (e) as probability density of the log-normal size distribution with added particle diameters obtained from the different PDF structural models and (f) structure of the Ir_192_ decahedral cluster.

Having established the overall evolution in atomic structure in the potential steps, we now analyze the PDFs in detail. We first address the structure of the reduced, metallic Ir nanoparticles. The PDF extracted at 0.2 V vs RHE is characteristic of a metallic nanoparticle with pair distances extending to ca. 12 Å (see Figure 2a). Bulk metallic iridium is known to crystallize in the face-centered cubic (*fcc*) crystal system, and we first test if the nanoparticles adopt the same structure. The extracted PDF can however not be fully described by a *fcc* model, see the fit result in Figure 2a (*R*_w_ = 0.34). Certain peaks appear in the PDF, e.g., at ca. 5 Å (area highlighted in gray in Figure 2a), which do not correspond to pair distances for the bulk *fcc* structure.

It is well-known that small metallic nanoparticles can differ in structure from their bulk counterparts and take, e.g., multitwinned structures forming, e.g., decahedral^62−64^ or icosahedral particles.^65^ It has furthermore been shown that osmium-based nanoparticles can form both *fcc* and hexagonal close-packed (*hcp*) structures,^66^ and in other systems, stacking faults have been seen to be dominating for small nanoparticles.^67^ PDF analysis allows us to characterize and distinguish between such structures, as it is sensitive to the local atomic arrangements.^59−61,68−70^ Recently developed tools allow to automation of the analysis of PDFs from metallic nanoparticles to identify the best-fitting model.^68,71,72^ Here, we first applied our newly developed method, DeepStruc, which uses deep learning to solve the structure of monometallic nanoparticles of up to 200 atoms from the PDF data.^72,73^ As seen in Figure S14, DeepStruc suggests that the metallic Ir nanoparticles take either a *hcp* or a decahedral structure. When fitting a *hcp* cluster model (Figure S15), the peak at ca. 5 Å is included in the simulated PDF, which was absent when using the *fcc* model. The *hcp* model is, however, also not able to describe all the distances present in the PDF data (*R*_w_ = 0.33). A better fit is achieved with a two-phase model including both a *fcc* and a *hcp* phase (*R*_w_ = 0.28), which is shown in Figure 2b. Mixtures of crystalline *hcp* and *fcc* Ir phases under electrochemically reductive conditions have been reported.^74^ Also, a two-phase model of *fcc* and *hcp* structures has previously been used for PDF modeling of close-packed structures that contain stacking faults.^67^ The agreement between data and the two-phase model could thus indicate that the nanoparticles consist of defective, closed-packed structures. However, the model assumes a spherical shape factor, and the crystallite sizes refine to 9 Å for the *fcc* and 11 Å for the *hcp* phase. Therefore, to further analyze the nanoparticle structure, their particle size was analyzed with SAXS as well. We obtain a mean particle size of 14 Å by fitting a model of polydisperse spherical particles with a log-normal distribution (polydispersity of PD = 0.2) and a power-law to the SAXS curve as shown in Figure 2d and Table S1. The log-normal size distribution of the polydisperse spheres model fit to the SAXS data is presented in Figure 2e. The SAXS results thus do not agree with the two-phase *fcc* and *hcp* model.

We now consider decahedral structures, which were also suggested by DeepStruc. Decahedral particles are constructed from *fcc*-shaped crystals separated by twin boundaries, resembling stacking faults.^72^ Decahedral metal nanoparticles are readily obtained in many systems,^75^ and the formation of decahedral Ir nanoparticles has been reported via a similar synthesis route.^76^ To validate our modeling approach further, we used a brute-force structure-mining algorithm^71,72^ as presented in the SI in Table S2 and Figure S16. This algorithm also suggests decahedral particles as best-fitting structural models, and the best fit when modeling the experimental PDF with an Ir_192_ decahedral particle (Figure 2f). The fit result is presented in Figure 2c (*R*_w_ = 0.31). Compared to the two-phase *fcc*/*hcp* model, the decahedral Ir_192_ cluster has a diameter (ca. 16 Å) that agrees better with the particle diameter obtained in the SAXS measurement, while the fit obtains a comparable *R*_w_ value and uses significantly fewer parameters in the fitting procedure.

The match between the PDF and SAXS size analyses is highlighted when comparing the diameters obtained from the PDF fits for the different structural models to the particle size distributions obtained from the SAXS model, as shown in Figure 2e. It can be expected that the data can be even better described by introducing a distribution of differently sized decahedral particles or additional stacking faults in the metallic nanoparticles. Our combined PDF and SAXS analysis of the Ir nanoparticles at 0.2 V vs RHE shows that the structure can be best described as decahedral-shaped metallic nanoparticles.

### Electrochemical Formation of Iridium Oxide before OER

**Figure 3:**
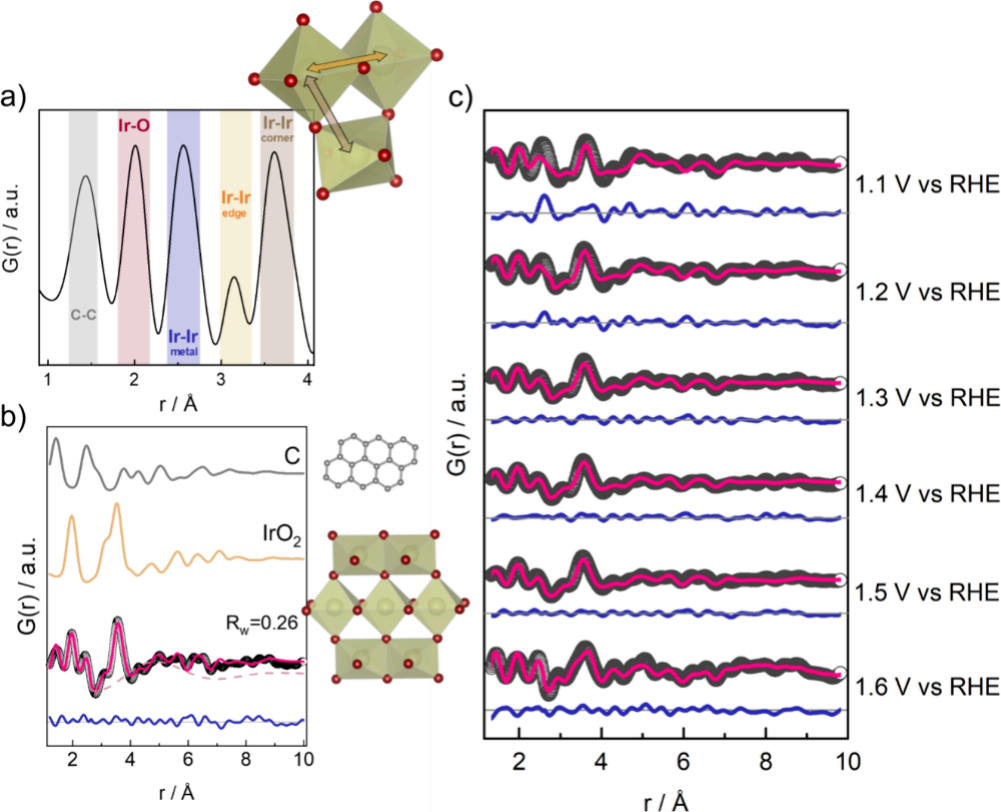
(a) Local range (1–4 Å) of the PDFs of the electrochemically formed iridium oxide with remaining metallic character in the particle core (1.1 V vs RHE) and illustration of a rutile structural motif of edge- and corner-sharing [IrO_6_] octahedral units and (b) fit of a rutile-structure cluster model to the PDF data collected at 1.5 V vs RHE, showing the individual component contributions to the fit as well as (c) overview of the fits obtained for the cluster model for all potentials from 1.1 to 1.6 V vs RHE.

We now analyze the oxide formation of Ir nanoparticles under electrochemical conditions in detail. From a potential of 1.1 V vs RHE, Ir oxide formation is obvious. The local range of the PDF obtained at this potential is shown in Figure 3a. In the PDF, the Ir–O peak at ca. 2 Å (highlighted in red) evidences the oxidic character of the material. Further peaks are seen at ca. 3.2 and 3.6 Å, highlighted in orange and brown in Figure 3a. These peaks agree well with Ir–Ir distances in iridium oxide. Based on reported crystalline iridium oxide structures,^77,78^ we can assign the peak at ca. 3.2 Å to Ir–Ir in neighboring edge-sharing [IrO_6_] octahedra (orange arrow in the structure cut-out), while the one at 3.6 Å corresponds to Ir–Ir in corner-sharing [IrO_6_] octahedra (brown arrow in the structure cut-out). Simulated PDFs of different iridium oxide structures are presented in Figure S17. Based on the ratio of corner-sharing to edge-sharing Ir octahedra, we can further characterize the atomic structure of the formed iridium oxide. Hollandite-type iridium oxide motifs, which have been found in low-crystalline iridium oxide samples that are highly OER active,^77^ would be characterized by an equal number of corner-sharing to edge-sharing [IrO_6_] octahedral units. As seen in Figure S11, this leads to two Ir–Ir peaks of similar intensity. However, in our experimental PDF, the corner-sharing peak is more intense than the edge-sharing peak. This ratio of corner-sharing to edge-sharing Ir octahedra indicates the presence of a rutile-type oxide, where one [IrO_6_] octahedron has two and eight edge-and corner-sharing neighbors, respectively, see structure cut-out in Figure 3.

The PDF contains very few peaks at distances larger than 8 Å. However, the SAXS scattering curves obtained at oxidative potentials show only minor variations (Figure S18), corresponding to average mean nanoparticle sizes of 14–15 Å (extracted from the SAXS fits at 1.1 and 1.3 V vs RHE, see Table S1 and Figure S19). This indicates that the absence of peaks at higher distances in the PDF is related to a lack of long-range order, rather than a reduction in nanoparticle size: While the particles locally take a rutile-like structure composed of edge- and corner-sharing [IrO_6_] units, the structural coherence is significantly smaller than the nanoparticle size.

The PDF also shows that at 1.1 V vs RHE, the iridium metal is not completely oxidized, as the edge-sharing Ir–Ir distance of ca. 2.7 Å (blue highlight in Figure 3a) is still visible. Compared to the partially oxidized nanoparticles at open circuit potential, we find no significant differences in the iridium oxide structure formed at 1.1 V vs RHE, as seen in Figure 1.

Having established that the electrochemically oxidized iridium nanoparticles show structural similarities to rutile, we now attempt modeling the PDFs with the rutile structure. We note here that the *operando* PDF still contains a carbon contribution, which is a result of the data treatment and the structural changes taking place in the carbon during the experiments. Before Fourier-transforming the total scattering data to obtain the PDF, the signal from the carbon support was subtracted to isolate only the contribution from the Ir nanoparticles. Despite our efforts, achieving a complete background correction of the carbon support proved difficult for the *operando* measurements. This difficulty is mainly related to the fact that the carbon support changes during the measurement, i.e., it becomes oxidized. We observed an additional peak at around 1.4 Å in the PDF, which corresponds to the C–C distance of the carbon support material. The PDF of the carbon support is shown in Figure S20. We note that a second C–C distance from the support is expected at 2.5 Å, which is however hidden underneath the Ir–Ir distance of the metal. The superposition of the two signals leads to an apparent shift of the Ir–Ir metal distance to smaller distances, which we regard as an artifact from the insufficient background correction of the carbon support. Similarly, the peak originating from the Ir–Ir corner distance is convoluted with the signal from the C–C distance at around 3.7 Å. We, therefore, include a carbon phase in the model to improve the fitting of the PDFs for the iridium oxide phase. The fits obtained and the details of this fitting approach are given in the Supporting Information (Figure S21). When fitting the PDF data to a rutile model, we see some agreements between data and fit, but the model is not able to completely describe the data, and the relative peak intensities are not well matched.

### Rutile-Oxide Cluster to Describe the Iridium Oxide Structure

To obtain more quantitative insights into the structure of the electrochemically formed iridium oxide, we construct a series of cluster models based on the rutile structure. A similar approach was previously used to describe the domain structure of amorphous iridium oxide films formed by electrodeposition.^79^ We fitted the different cluster models to the *operando* PDF collected at 1.5 V vs RHE, as demonstrated in Figure S22. The cluster model, which gives the best description of the data, is shown in Figure 3b and is a planar cut-out of seven [IrO_6_] octahedral units from the rutile oxide crystal structure. The longest Ir–Ir atomic pair distance in the cluster model is 7 Å. With this rutile-based cluster model, we can describe the PDF data collected from 1.0 to 1.6 V vs RHE, as shown in Figure 3c. Details of the refinements are included in the SI, Table S3. The structure model is cut directly from the rutile structure, and we only refined a scale factor, atomic displacement parameters (ADPs) for Ir and O, and an isotropic cluster expansion/contraction factor, which means that the atomic arrangement in the cluster is not modified during the refinement.^68^ To account for residual carbon contributions, we included a single graphite sheet with a diameter of 10 Å in the model. Our model further included a damped sinusoidal function. Here, we use the wave function, which has also been used to describe solvent restructuring.^80^ The contribution of the individual components of the model to the PDF fit is shown in Figure 3b. The need for such a wave function to properly describe the PDF may relate to the correlation between IrO_*x*_ domains in the materials. However, the challenging nature of the *operando* data and difficulties in background subtraction means that no further analysis of this effect will be done.

The metal Ir–Ir contribution present in the data collected at 1.0 V vs RHE leads to a residual curve, which has a prominent peak at 2.7 Å. From a potential of 1.3 V and higher, we do not observe this metal contribution in the residual curve. Our PDF analysis combined with the results from the SAXS fit, thus, shows that the formed iridium oxide can be described as 1.5 nm nanoparticles with no long-range order, but with a local rutile structure motif.

### Changes in Catalyst Structure When Going to OER Conditions

**Figure 4:**
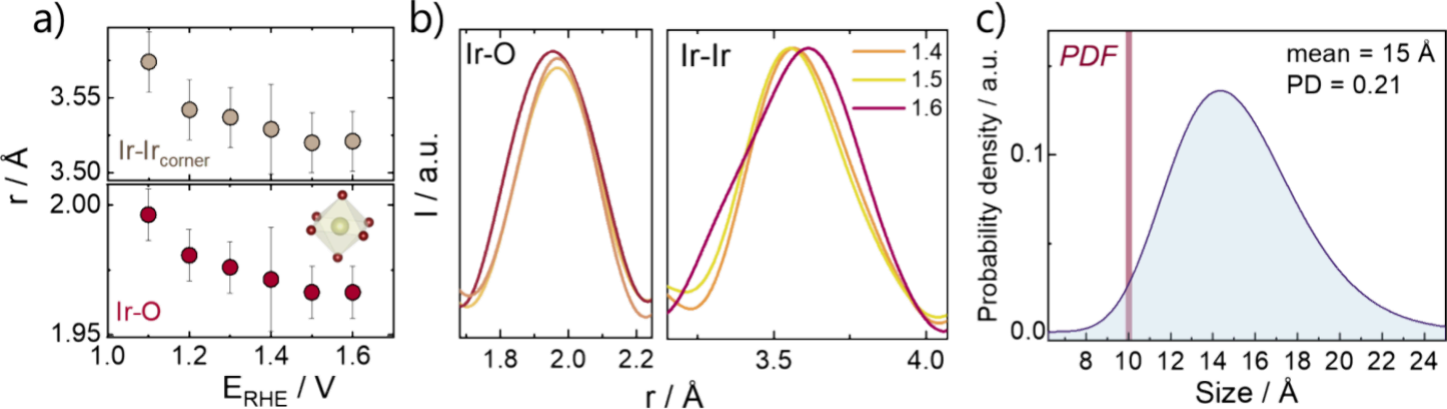
(a) Evolution of Ir–Ir distance of corner-sharing octahedra and the weighted Ir–O distance obtained for the rutile cluster fitting approach as a function of applied potential. (b) Zoom in on the Ir–O and Ir–Ir pair distances of the PDFs collected at 1.4, 1.5, and 1.6 V vs RHE. (c) Log-normal size distribution of the SAXS model fit to the 1.5 V vs RHE data, with a mean size of 15 Å and a polydispersity (PD) of 0.2. The diameter of the rutile-structure cluster model fit to the PDF data collected at 1.5 V vs RHE is indicated with the red line marked PDF.

When we applied increasingly oxidative potential steps, small changes in the atomic structure of the nanoparticles can be observed in the PDFs. Figure 4a (bottom panel) displays the Ir–O distance of the [IrO_6_] octahedra extracted from the PDF fits plotted in Figure 3. As the structure model was cut out directly from the IrO_2_ crystal structure (ICSD 84577),^81^ it contains two distinct Ir–O distances of 2.00 Å (coordination number CN = 4) and 1.96 Å (CN = 2) for bulk IrO_2_. To illustrate the Ir–O pairs we show a CN-weighted average of the two bonds in Figure 4a. Here, our refinements show a shortening of the Ir–O pairs with higher anodic potential. This corresponds to a contraction of the Ir–O bonds when the iridium becomes progressively more oxidized.

Similarly, the Ir–Ir distance of ca. 3.6 Å in corner-sharing [IrO_6_] becomes shorter, when the potential is increased, indicating a contraction of the iridium oxide structure at higher potentials, which agrees with more oxidized iridium (Figure 4 a, upper panel).

The same trend of Ir–O and Ir–Ir bond contraction is observed for the repeated measurement of a second catalyst film (see Figure S23), confirming that this is a general effect. The extracted distances for the two independent measurements differ only slightly. The deviations of absolute interatomic distances extracted from the PDFs are likely due to variations in the alignment of the sample when measuring in *operando* conditions, which lead to slight differences in sample-to-detector distance in the two independent experiments. Therefore, the investigation of trends in the evolution of bond and pair distances is more relevant in this study.

We also performed *operando* extended X-ray absorption fine structure (EXAFS) experiments. Analysis of these data allows us to further analyze the local atomic arrangement of the iridium oxide structure under applied potential, to confirm the trends observed from the *operando* PDF experiment. Details of the EXAFS experiment are given in the SI (Table S4, Figures S24–S26). We observe a similar Ir–O bond contraction from 1.98(2) to 1.95(2) Å during electrochemical iridium oxidation. At the OER potential (1.5 V vs RHE), the feature corresponding to Ir–O in the FT-EXAFS broadens significantly and a shorter iridium oxygen bond becomes apparent (Figure S25). In the modeling of the EXAFS, an additional Ir–O distance of 1.93(1) Å is necessary to describe the data. This finding of a shorter Ir–O bond that is present during OER agrees with the observed broadening of the Ir–O peak at 1.6 V vs RHE in the *operando* PDF (cf. peak at 2 Å in Figure 1a). The short Ir–O bond distance seen at 1.5 V vs RHE could indicate the presence of an Ir^5+^ intermediate, as previously described by Diklic et al.,^12^ or the formation of an O-^–^ intermediate, as found by Pfeifer et al.^10,29^ Similarly, Lebedev et al.^82^ identified a shorter Ir–O bond distance under OER conditions as an Ir(V)=O intermediate for single-atom Ir catalysts during OER by in situ EXAFS. The good agreement between the Ir–O coordination of several Ir-based OER catalysts synthesized by different approaches could indicate that this is a general feature of the active catalyst structure. We note here that all the X-ray techniques employed to study the atomic structure of the OER active nanoparticles are bulk techniques. Due to their small nanoparticulate nature, we can, however, extract some information about the surface structure.^83,84^ For 1.5 nm Ir nanoparticles, up to 60% of iridium atoms are surface atoms, as determined for Ir nanoparticles on ITO support.^85^ The structural variations evident under OER conditions can, therefore, be related to changes in surface structure.

The Ir–Ir bonds extracted from the EXAFS fits also contract upon oxidation, but are generally slightly shorter than those observed in the PDF. The extracted Ir–O and Ir–Ir bond distances from modeling the *operando* PDF and EXAFS data are shown in Figure S27. The slight differences may be due to alignment uncertainties or could be rooted in experimental differences between the two *operando* cells. The same general trend of bond contraction has previously been observed in an *operando* EXAFS study of ultrasmall Ir nanoparticles of ca. 2 nm diameter, prepared by the Adams fusion method when applying potential steps in the same potential region.^30^ Due to the different experimental set-ups used to collect the *operando* X-ray scattering and X-ray absorption data, we discuss only trends observed in the experiments and refrain from comparing the XAS and PDF experiments in more detail.

### Structural Disorder in the OER Active Ir Oxide

After having discussed the general trends of electrochemical iridium oxide formation, we now analyze the structure of the OER active oxide, i.e., at 1.6 V vs RHE, in more detail. In general, no substantial changes in oxide structure occur when a significant OER current is measured, as seen in Figure 3. The local coordination of a rutile-type oxide with edge and corner-sharing [IrO_6_] octahedra is still evident and no long-range order is visible. However, at the potential of 1.6 V vs RHE, peak broadening for both the Ir–O and Ir–Ir nearest neighbors is observed (Figure 4b), which can be interpreted as increasing mobility of the atoms in the structure at higher oxygen evolution currents (steady-state current *i* = 130 mA/mg_Ir_). This is also reflected in the increased ADPs obtained in the PDF modeling (Table S3). The ADPs for O refined to 0.015 Å^2^ compared to 0.013 Å^2^ at 1.4 V vs RHE, and to 0.014 Å^2^ compared to 0.009 Å^2^ for Ir, respectively. We also observed increased Debye–Waller factors extracted from the EXFAS fit at 1.5 V vs RHE for Ir (0.009 Å^2^) and O (0.008 Å^2^), compared to 0.006 Å^2^ for Ir and 0.005 Å^2^ for O at 1.3 V vs RHE (see Table S4). This highlights that under operating conditions, the OER active iridium oxide catalyst is characterized by a large amount of structural flexibility.

To further study the disordered nature of the electrochemically formed iridium oxide, we again compared the sizes extracted for the nanoparticles at 1.5 V vs RHE from the SAXS data with those from the corresponding PDF data. Fitting a polydisperse sphere model to the SAXS data (Figure S17), we obtained a mean particle size of 15 Å in diameter, i.e., showing no growth of the particles when going to OER conditions. The SAXS curves do not show a contribution from a structure factor *S*(*q*) which would indicate aggregation of nanoparticles. The log-normal size distribution of the particles is shown in Figure 4c. The rutile structured cluster fit to the PDF extends to only 10 Å. The much smaller spherical diameter of the iridium oxide obtained from the PDF (highlighted in red in Figure 4c) compared to the SAXS model indicates an iridium oxide with only short-range atomic order.

**Figure 5:**
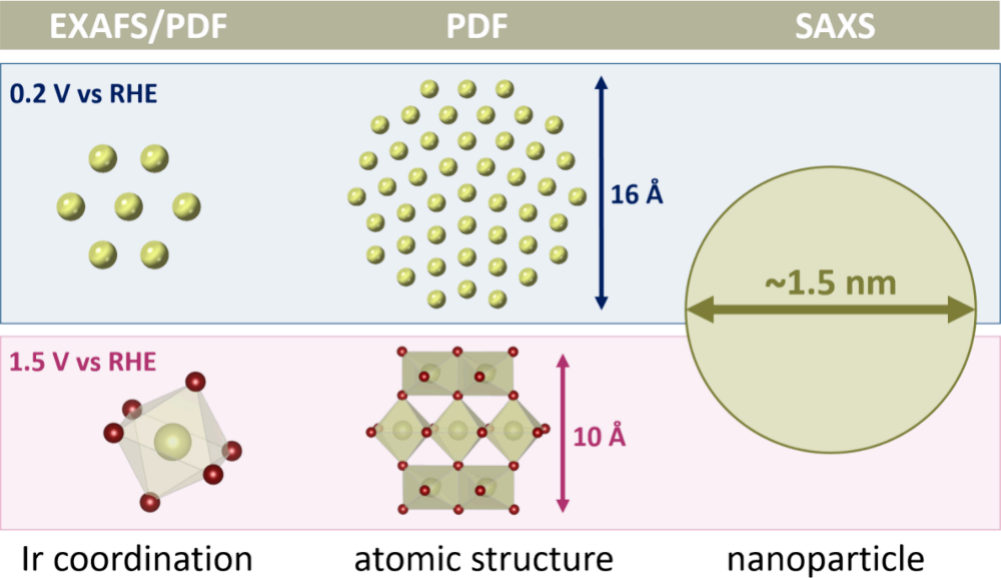
Sketch illustrating the information that can be extracted by *operando* X-ray PDF and SAXS on the structure of the electrochemically formed iridium oxide, which is active for OER. While PDF can provide information on the very local Ir–O and Ir–Ir coordination similar to EXAFS, PDF can also provide information on the arrangement of metal polyhedra within the nanoparticle. Combined with morphological information from SAXS, a complete picture of the atomic structural arrangement in the iridium oxide nanoparticle is obtained.

Only by combining *operando* SAXS, PDF, and EXAFS analysis, we can fully characterize the structure of the electrochemically formed OER active oxide. The information that is obtained from the different techniques is sketched in Figure 5 for both the metallic and OER active oxide structure. We use SAXS to extract the nanoparticle size of the OER active iridium oxide and PDF analysis to determine the atomic arrangement inside the particles, while EXAFS gives further insight into the local structural arrangement and oxidation state of iridium during OER. By combining these complementary techniques, we show that the metallic nanoparticles are decahedrally shaped. When electrochemically oxidized, an iridium oxide that lacks long-range order forms. The oxide consists of Ir–O octahedral units that are connected in a rutile-like fashion in small clusters ordered up to max. 10 Å, while the particle itself is much larger with a 15 Å diameter. Under OER conditions, the structure becomes even more disordered. Such structural flexibility and disorder have been related to higher OER activity, also, e.g., when comparing electrochemically prepared iridium oxide to calcined crystalline rutile IrO_2_.^10^ The formation of amorphous Ir oxo-hydroxide shells under OER conditions has been reported for different Ir-based OER catalysts of similar particle sizes in several studies.^30,85−87^

## Conclusions

We investigated the structural dynamics of ultrasmall iridium nanoparticles exposed to an electrocatalytic OER environment. Employing a combination of *operando* techniques of X-ray total scattering and PDF analysis with analysis of *operando* SAXS data, we can extract valuable insights into the atomic arrangement and morphology of the Ir nanoparticles before and during OER. Despite the challenges in *operando* experiments, including accounting for the changing background signal during experiments, we are able to extract quantitative structural information on the catalyst structure. We first reduce any surface oxide to study the structure of the metallic Ir nanoparticle under potential control, but nonreactive conditions. Interestingly, the atomic structure of the Ir nanoparticles cannot be fully described by an *fcc* phase but shows additional *hcp*-like features. Further analysis of the PDF reveals that the metallic nanoparticles at 0.2 V vs RHE are most likely Ir_192_ decahedral clusters. The diameter of the Ir_192_ cluster is comparable to the mean particle diameter obtained from the SAXS analysis (14 Å). When applying oxidizing potentials, we identified a clear formation of an iridium oxide with no long-range order. The local structure of the electrochemically formed Ir oxide contains both corner- and edge-sharing Ir–O octahedra, similar to a rutile oxide, albeit displaying limited structural order. Increasing the applied potential further, we observed that the residual metal is gradually oxidized and that the Ir–O bond length of the oxide structure contracts. At relevant OER current densities, a discernible rise in atomic mobility became evident, notably reflected in large ADP values for Ir and O.

Using *operando* SAXS analysis, we determine the diameter of the iridium oxide nanoparticles to be consistently around 15 Å at 1.5 V vs RHE. Given that the rutile-structure cluster model that describes the PDF only extends to 10 Å for iridium oxide, we infer that the atomic structure of the OER-active oxide exhibits considerable disorder, yet, contains local rutile-like structural motifs. In conclusion, our findings underscore that *operando* PDF analysis is a powerful tool for extracting information on the atomic arrangement of ultrasmall nanoparticles. When combined with *operando* SAXS and EXAFS analysis, this approach provides a comprehensive understanding of the disordered nature of the OER active phase that is formed in a real electrochemical environment.

## Data Availability

The experimental data collected for this manuscript are accessible through the ESRF for proposal CH 6227 via the following doi: 10.15151/ESRF-ES-790330698.

## Supporting Information Available

Experimental details of the operando experiments and data modeling approaches, electrochemical analysis, TEM micrographs, additional PDF analysis, XANES data, and EXAFS fitting (PDF)
