# Supplementary material for: Monitoring the Structural Changes in Iridium Nanoparticles during Oxygen Evolution Electrocatalysis with Operando X-ray Total Scattering
Source: J Am Chem Soc. 2024 Sep 30;146(40):27517–27. doi: 10.1021/jacs.4c08149 (PMC11468871; doi:10.1021/jacs.4c08149)
Supplement: Supplementary file 1 — ja4c08149_si_001.pdf [file ja4c08149_si_001.pdf]

Supporting Information

Monitoring the structural changes in iridium nanoparticles during oxygen evolution electrocatalysis with *operando* X-ray total scattering

Rebecca K. Pittkowski[1*], Stefanie Punke[1], Andy S. Anker[1], Aline Bornet[2], Nicolas Pierre Louis Magnard[1], Nicolas Schlegel[2], Laura G. Graversen[1], Jonathan Quinson[3], Alexandra Dworzak[4], Mehtap Oezaslan[4], Jacob J. K. Kirkensgaard[5,6], Marta Mirolo[7], Jakub Drenc[7], Matthias Arenz[2*], Kirsten M. Ø. Jensen[1*]

[1] Department of Chemistry, University of Copenhagen, Universitetsparken 5, 2100 Copenhagen, Denmark

[2] Department of Chemistry, Biochemistry and Pharmaceutical Sciences, University of Bern, Freiestrasse 3, 3012 Bern, Switzerland

[3] Biological and Chemical Engineering Department, Aarhus University, 40 Åbogade, 8200 Aarhus, Denmark

[4] Technical Electrocatalysis Laboratory, Institute of Technical Chemistry, Technische Universität Braunschweig, 38106 Braunschweig, Germany

[5] Niels Bohr Institute, University of Copenhagen, Universitetsparken 5, 2100 Copenhagen, Denmark

[6] Department of Food Science, University of Copenhagen, Rolighedsvej 26, 1958 Frederiksberg, Denmark

[7] ESRF - The European Synchrotron, 71 Avenue des Martyrs, 38000 Grenoble, France

*corresponding authors: rebecca.pittkowski@chem.ku.dk, matthias.arenz@unibe.ch, kirsten@chem.ku.dk

Contents

Section 1 - Materials and Methods 3
- Catalyst and electrode preparation 3
- Transmission electron microscopy 3
- In-house electrochemical measurements 3
- *Operando* scattering experiments 4
- Scan through the catalyst layer 6
- X-ray absorption spectroscopy (XAS) 6
- PDF Modeling 7
- SAXS Modeling 8

Section 2 – Experimental Supporting Information 11
- TEM characterization 11
- Cyclic voltammograms 12
- *Operando* electroreduction of surface oxide 13
- Oxygen evolution Tafel plot 14

Reproducibility of the measurements 15
- Total scattering in reciprocal space 15
- Total scattering in real space 16

Structure of metallic nanoparticles after electroreduction 19
- Decahedral clusters 21

Structure of electrochemically formed iridium oxide 25

Operando EXAFS analysis 33

References 37

Section 1 - Materials and Methods

Catalyst and electrode preparation

Ir nanoparticles (NPs) immobilized on carbon support (Ir/C) were synthesized using the same protocol described by Bornet *et al*.[1] To obtain Ir NPs, a mixture solution containing 2 mL of 20 mM $IrCl_3$ (99.8% metal basis, Alfa Aesar) dissolved in ethanol (EtOH, EtOH absolute, VWR Chemicals) and 7 mL of 57 mM NaOH/EtOH (NaOH: Hänseler) solution was allowed to react in an oil bath at 85 °C for 10 min under reflux conditions and constant stirring (350 rpm). Once the NPs were synthesized (brown solution color), they were immobilized on carbon Ketjen Black (EC-300J, Fuel Cell Store) support. To do so, the support was first dispersed in EtOH (1:2 mass (support): volume (EtOH)) for 4 min using a horn sonicator (pulse: 1 s on / 1 s off, amplitude 30%, Q500 sonicator, QSONICA sonicators) and then poured into the freshly synthesized NPs. The final dispersion was further sonicated for 10 more min under the same conditions.

EtOH was evaporated using a rotary evaporator (120 rpm, room temperature (RT), 5 °C cooling system, RC 600, knf) under constant sonication. The catalyst powder was further dried using a rotary evaporator (25 rpm, water bath at 85 °C, 5 °C cooling system, 30 mbar, 4 h) on the next day. This second step allows the removal of undesired side products.

An ink with a concentration of 250 μg_{Ir} mL^{-1} was prepared by dispersing the as-synthesized catalyst in a 3:1 volume ratio of ultra-pure water (MilliQ-system, 18.2 MΩ.cm) and isopropanol (IPA, HPLC grade, VWR Chemicals). To improve the stability and enhance the homogeneity of the ink,[2] 70 µL of 1 M KOH (Hänseler) was added per 60 mL of ink. 10 wt.% Nafion dispersion in H_2O (D1021, Fuel Cell Store) with respect to the catalyst (NPs and support) was added and the ink was sonicated for 5 min at RT.

The electrodes were prepared by vacuum filtration of the Ir/C catalyst ink onto a gas diffusion layer (GDL) coated with a C-based microporous layer (MPL) (Freudenberg H23C8, 0.230 mm thick, Fuel Cell Store). The coated GDL (Ø 3.7 cm) was placed between a sand core filter and a glass reservoir of a vacuum setup. 8.6 mL of the 250 μg_{Ir} mL^{-1} ink was diluted with 30.1 mL of IPA and 4.3 mL of H_2O. By this approach, after filtration, a homogenous catalyst layer of circa 10 µm depth was obtained with a nominal loading of 200 μg_{Ir} cm^{-2}.

Transmission electron microscopy

The samples for TEM analysis were prepared by drop casting the as-prepared Ir nanoparticles or diluted Ir/C supported catalyst in methanol or ethanol onto carbon-coated copper TEM grids (Quantifoil). A Jeol 2100 operated at 200 kV and a Jeol 3000F operated at 300 kV were used to characterize the as-prepared colloids. A Tecnai Spirit operated at 80 kV was used to characterize the supported particles. The size distribution was obtained using the ImageJ software and retrieved by estimating the diameter of at least 100 individual nanoparticles.

In-house electrochemical measurements

An electrochemical cell, named GDE setup, in a three-electrode configuration was used to measure cyclic voltammograms of the Ir/C catalyst (50 wt.%) deposited on the GDL (200 $\mu g_{Ir}/cm^2$). The catalyst-loaded GDL with a 3 mm diameter was used as the working

electrode with a platinum mesh as the counter electrode. All potentials were measured in reference to a reversible hydrogen electrode (RHE) as the reference electrode. A pre-treated Nafion membrane (2 cm diameter, Nafion 117, 183 µm thick, Fuel Cell Store) was used to separate the catalyst from the electrolyte. The activation is described in detail in the literature.[1] The measurements were performed with a potentiostat (ECi – 242 from Nordic Electrochemistry ApS), controlled with the software EC4DAQ in version 2.44. Humidified (with Milli-Q water, 18.2 MΩ.cm) oxygen (Alphagaz 1, 45) was continuously flown through the setup during the measurements, with a flow rate between 50 and 60 $mLmin^{-1}$. As the electrolyte, 4 M $HClO_4$ (prepared from 70% $HClO_4$ (ACS reagent, Sigma Aldrich) was used in the upper polyetheretherketone (PEEK) compartment of the setup. The electrolyte was preheated to max. 7° C above the desired temperature of 60° C. An aluminum-made faradaic cage was used in the experiment that was preheated to 60° C using a thermocouple-controlled heating plate. The cyclic voltammograms (CVs) were recorded between 1.2 and 1.6 V vs RHE at a scan rate of 10 mVs^{-1}.

***Operando* scattering experiments**

The experiments were performed in a transmission diffraction electrochemical cell where the microfocused X-ray beam is introduced in the plane of the catalyst layer. The cell design has been previously described by Martens *et al*.[3] The transmission diffraction electrochemical cell is run in a three-electrode set-up with a 1 cm diameter circular working electrode (Ir/C functionalized GDL), a platinum counter electrode, and a leakless Ag/AgCl (eDAQ) reference electrode. The cell is shown in **Figure S1**. The electrolyte used in the experiments was 0.5 M $HClO_4$, prepared from 70% $HClO_4$ (ACS reagent, Sigma Aldrich) diluted with ultra-pure water (MilliQ-system, 18.2 MΩ.cm). The electrolyte was pumped through the cell at a flow rate of 0.2 ml/min. Before and after each potential step protocol, the reference potential of the Ag/AgCl electrode was measured against a freshly prepared reversible hydrogen electrode (RHE) to ensure the stability of the reference electrode potential. All potentials are converted to the RHE scale. The solution resistance of the cell was determined by impedance spectroscopy and the IR drop was compensated at 85% during the measurements.

The experimental protocol consisted of stepping the potential continuously. After holding the potential at open circuit potential (OCP) for 10 minutes, a series of potential holds (0.2, 0.5, 1.1, 1.2, 1.3, 1.4, 1.5, and 1.6 V vs RHE) of 10 min duration each was applied. Scattering data were collected after the initial two minutes of each potential hold.

X-ray measurements were performed at beamline ID31 at the European Synchrotron Radiation Facility (ESRF). The set-up with the electrochemical cell installed with the detector in position for total scattering measurements is shown in **Figure S1.**

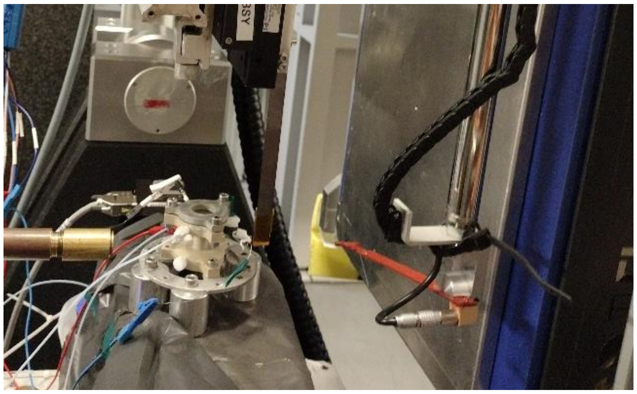

Figure S1 Set up used in the *operando* scattering experiments at ID31 at the ESRF with the detector in total scattering mode with a low sample-to-detector distance.

A wavelength of 0.16531 Å was used for all experiments. The beam was focused to achieve a vertical beam size of 6 μm. The collected 2D scattering images were azimuthally integrated with the PyFAI software package.[4] A height scan (z-scan) through the catalyst layer was performed to collect scattering data throughout the whole catalyst-containing layer as well as from the carbon support and the electrolyte in the cell,[5] as shown in **Figure S2**. The z-depths corresponding to the carbon support in the cell (highlighted with a blue line in **Fig. S2**), as well as the electrolyte in the cell (highlighted with a pink line in **Fig. S2**), were used for background subtraction.

X-ray total scattering data were measured using a Dectris Pilatus3 X CdTe 2M detector, at a detector-to-sample distance of circa 19 cm. A CeO_2 powder standard was used to calibrate the exact geometry. Total scattering data were collected with an exposure time of 30 seconds, and a total of three images from the collection heights with maximum iridium content were averaged for each data point. The X-ray total scattering data were Fourier transformed to obtain the pair distribution functions (PDFs) using xPDFsuite.[6] The following parameters were used for data reduction: Q_{min} = 1.2 Å^{-1}, Q_{max} = 17.5 Å^{-1}, $Q_{maxinst}$ = 25 Å^{-1} and r_{poly} = 0.9 Å.

Small Angle X-ray scattering (SAXS) data were collected with a Dexela 2923 Detector, at a detector-to-sample distance of circa 8.2 m. The SAXS data were recorded with three seconds of exposure time. The sample-to-detector distance was calibrated using an AgBh standard. An evacuated flight tube was used to reduce parasitic air scattering in the SAXS geometry.

Scan through the catalyst layer

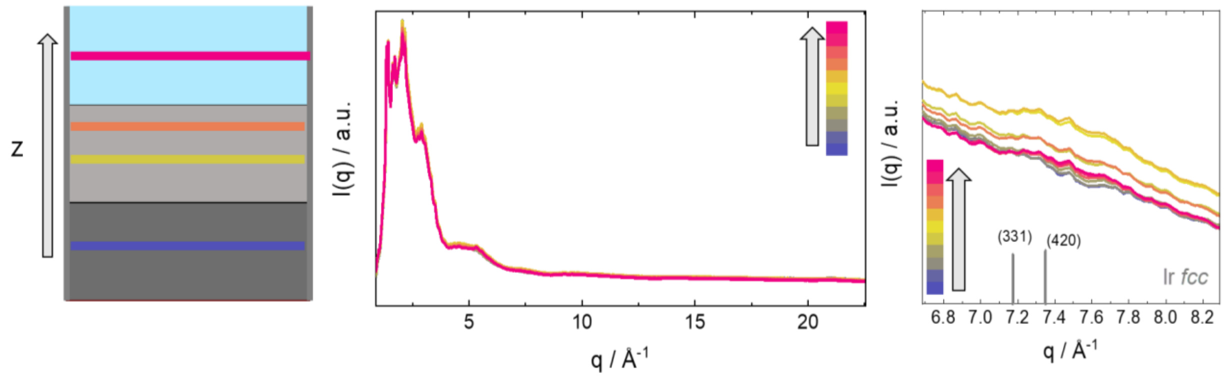

Figure S2 Sketch of the catalyst layer in the cell (on the left), which is probed with the X-ray beam at different heights in the z-direction as indicated by the grey arrow. The light blue area at the top depicts the electrolyte in the cell, the light grey area corresponds to the Ir-containing part of the GDL, while the dark grey area illustrates the carbon support of the GDL (C-MPL) without iridium particles. In the middle, the scattering patterns collected at the different z-positions are shown for the sample during a potential hold at 0.2 V vs RHE. The pattern is dominated by the scattering peaks resulting from the setup. On the right, we show a blow-up of the region around q = 7 - 8 $Å^{-1}$, where scattering from the metallic iridium dominates (Bragg reflections (331) and (420) of Ir *fcc*) largely without background contributions, which can be used to identify the iridium containing part of the functionalized GDL.

Challenges in operando X-ray total scattering experiments

Performing *operando* X-ray total scattering experiments is connected to a series of experimental difficulties. The alignment of the cell with respect to the detector is crucial, as even small differences lead to large variations, as the detector is very close to the *operando* cell to be able to measure to high q. As it is not possible to use an internal standard, uncertainties remain. Background subtraction of the different components present, such as the liquid electrolyte, catalyst support materials, and the cell itself, requires careful measurement of all scattering signals of the individual components, e.g. though a depth-scan trough the layer. This is further complicated by changes in the background contribution during the data collection, which can be related to changes in scattering signal with dissolution or intense bubble formation. In general, lower quality of the scattering data compared to capillary measurements are achieved, which is the result of limitations due to cell geometries. As different techniques, such as e.g. XAS, require other separate set ups, a comparison to other cells is often challenging.

X-ray absorption spectroscopy (XAS)

The *operando* XAS experiments were conducted at the ROCK beamline[7,8] of the Synchrotron SOLEIL. The beam current was 450 mA. The incident beam was collimated with a bending magnet (Ec = 8.65 keV) and a toroidal Si mirror with 50 nm Ir coating and monochromatized with a Si(111) monochromator. Two mirrors for harmonic rejection (1st flat, 2nd bendable) were tilted at 3 mrad with the stripe of Pd. The XAS measurements were performed at the Ir L_3 edge in transmission mode. The ionization chambers were

filled with pure nitrogen. The length of the ionization chamber for the incident X-ray intensity (I_0) was 188 mm. For the transmitted X-ray intensity after the sample and reference (I_1 and I_{ref}), the ionization chambers were both 328 mm long, respectively.
All the XAS data were processed and evaluated using the Demeter software package based on the IFEFFIT libraries.[9] Data processing included energy calibration of the raw data to the simultaneously measured Pt foil, data averaging, background correction, and normalization by the edge jump. The energy units (eV) were converted to photoelectron wave number k units ($Å^{-1}$) by assigning the photoelectron energy origin, E_0, corresponding to k = 0, to the first inflection point of the absorption edge. For the fitting of the extended X-ray absorption fine structure (EXAFS), the χ(k) functions were weighted with k^2. The k-range used for the Fourier transform was k = 3 - 13. The local structure parameters were refined from EXAFS functions by non-linear least square (NLLS) fitting in an R-space range of 1.3 – 3.2 Å. A Hanning window function was used in the Fourier transform. No phase correction was applied. The amplitude reduction factor of Ir, $S_0^2=0.89\pm0.04$ was refined from the first Ir-Ir shell of an Ir metal foil. To fit the EXAFS data, a three-shell model was constructed including the oxygen shell (Ir-O), the first metal oxide shell (Ir-Ir oxide), and the first metal-phase shell (Ir-Ir metal). The metal and oxide fractions were determined from the refined coordination numbers of the Ir-O and Ir-Ir scattering paths, considering the respective multiplicities of the oxygen and metal shells, following an approach described by Reksten *et al*. These were calculated as $f_{ox} = N_{(Ir–O)exp}/6$ and $f_{red} = N_{(Ir–Ir)exp}/12$, respectively.[10,11]

As an alternative method, the metal/oxide ratio was also determined by linear combination fitting (LCF) of the XANES data when using the respective XANES spectra of an Ir metal foil and an IrO_2 powder reference pellet as the components.[12] The LCF was performed with the Linear Combination Fitting tool available in ATHENA. The normalized μ(E) was fit in a range of -20 eV until + 50 eV around E_0. The edge position was allowed to vary but did not differ more than ± 1 eV from the initial value.

The electrodes used in the *operando* XAS experiment were prepared as described by Bizotto *et al.*[12], with a catalyst loading of circa 700 μg/cm^2 on the GDL. The electrodes were then mounted on a custom-made PEEK spectro-electrochemical transmission flow cell. The basic design is described by Binniger *et al*.[13] A graphite foil was used as a counter electrode and a leakless Ag/AgCl (eDAQ) as a reference electrode. Ar-saturated 0.1 M $HClO_4$ was flown through the cell with a flow rate of 1 mL min^{-1} controlled by a syringe pump.

PDF Modeling

Analysis of metallic Ir clusters.

For the Ir face-centered cubic (*fcc)* and hexagonal closest packed (*hcp)* models, the PDFs were modeled in PDFgui[6] using the *fcc* structure (space group $Fm\bar{3}m$) and *hcp* structure (space group $P6_3/mmc$). The refined parameters included a scale factor, the unit cell

parameters *a (fcc)* or *a* and *c (hcp)*, a spherical particle (Sp) diameter, δ_2 -parameter, and an isotropic atomic displacement parameter (ADP) value for Ir.

A brute-force structure-mining method[14,15] is tested, where libraries of clusters are automatically generated and refined against the provided experimental PDF. The clusters are generated by a structure builder in ASE,[16] and PDFs are generated using the Debye scattering equation for the intensity calculation and subsequently normalized, corrected and Fourier transformed to obtain the PDF. The structure refinement is performed between a simulated PDF and the experimental PDF by performing a least-square minimization. During structure refinement, an isotropic expansion coefficient, an isotropic ADP, and a single scale factor are refined. Structures with a good fit (here meaning a $R_w \leq 0.35$) are then individually fit to the experimental PDF in DiffPy-CMI.[17] Here the scale factor, isotropic ADP for Ir, the isotropic expansion/contraction parameter (zoom scale) r, and additionally a δ_2-parameter (Delta2) to account for correlated motion effects[6,18] were refined. The PDF refinement was performed in the r-range of 1.5 - 20.0 Å.

The iridium oxide was initially modeled in PDFgui[6,19] with a rutile-type IrO_2 (space group $P4_2/mnm$) and a carbon graphite structure (space group $P6_3/mmc$). The refined parameters were scale factors of both phases, unit cell parameters, isotropic APDs, and Sp diameters. The cluster fits of the rutile cut-out structure were performed with DiffPy-CMI as described for the metallic cluster fits. The model included the rutile cluster with 7 [IrO_6] octahedral units as well as a single sheet graphite cluster with a 10 Å diameter. We included a sine wave in the model.[20] All refined APD values are listed as U_{iso} throughout the paper ($U_{iso} = 1/8\pi^2 * B_{iso}$)

SAXS Modeling

The scattering signal from the catalyst has been corrected from the signal of the cell and the electrolyte as shown in **Figure S3** for the potential hold at 0.2 V vs RHE.

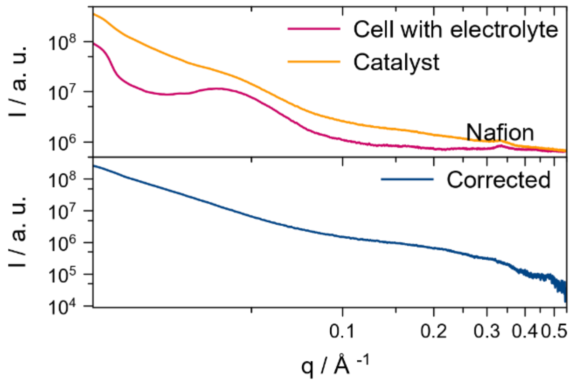

Figure S3 The SAXS patterns, from different parts of the cell, were collected during a potential hold of 0.2 V vs RHE. The small peak at around q = 0.35 $Å^{-1}$ can be ascribed to the Nafion ionomer used.

A model describing polydisperse homogenous spheres with a power-law background was fitted to the data.[5] The model can be written as the following expression:

$$I(q) = A_1 \int P_S(q,r)V(r)D(r)dr + A_2 q^{-n} + background$$

Where A_1 and A_2 are scaling constants, r is the particle radius, V(r) is the particle volume, n is the exponent of the power-law and P_S(q,r) is the spherical form factor given as

$$P_S(q,r) = \left[\frac{3\big(sin(qr) - qrcos(qr)\big)}{(qr)^3}\right]^2$$

and the number-weighted log-normal size distribution D(r) as

$$D(r) = \frac{1}{rPD\sqrt{2\pi}} exp\left[\frac{-\left(ln\left(\frac{r}{\mu}\right)\right)^2}{2PD^2}\right]$$

ith the polydispersity PD and the median µ of the distribution. This model is included in the SasView software package, version 5.0.5.[21] The resulting fit parameters are given in **Table S1.**

Table S1 Fit parameters obtained from fitting the model of a polydisperse sphere, as described above, to the SAXS data measured at 0.2, 0.5, 1.1, 1.3, and 1.5 V vs RHE. (*The polydispersity, PD, for the fit of the SAXS data measured at 0.5, 1.1, and 1.5 V vs RHE was set to the same value that was obtained in the fit of the SAXS measurement at 0.2 and 1.3 V vs RHE, respectively, because the fitted model with a freely variable PD was not able to describe the data).

**Potential vs RHE**	**A_1**	**Diameter $D_m = 2\ \mu$ (median)/ Å**	***PD***	**Diameter D_A (average)/ Å**	**A_2**	**n**
**0.2 V**	4.96E6 +/- 0.4E6	14+/- 1	0.2 +/- 0.01	14+/- 1	224+/- 10	3.38 +/- 0.04
**0.5 V**	5.24E6 +/- 0.4E6	14+/- 1	0.2*	14+/- 1	247+/- 10	3.36 +/- 0.04
**1.1 V**	2.42E6 +/- 0.4E6	15+/- 1	0.2*	15+/- 1	285+/- 10	3.34 +/- 0.04
**1.3 V**	4.89E6 +/- 0.4E6	14+/- 1	0.21 +/- 0.01	14+/- 1	180+/- 10	3.46 +/- 0.04
**1.5 V**	3.40E6 +/- 0.4E6	15+/- 1	0.21*	15+/- 1	197+/- 10	3.46 +/- 0.04

Section 2 – Experimental Supporting Information

TEM characterization

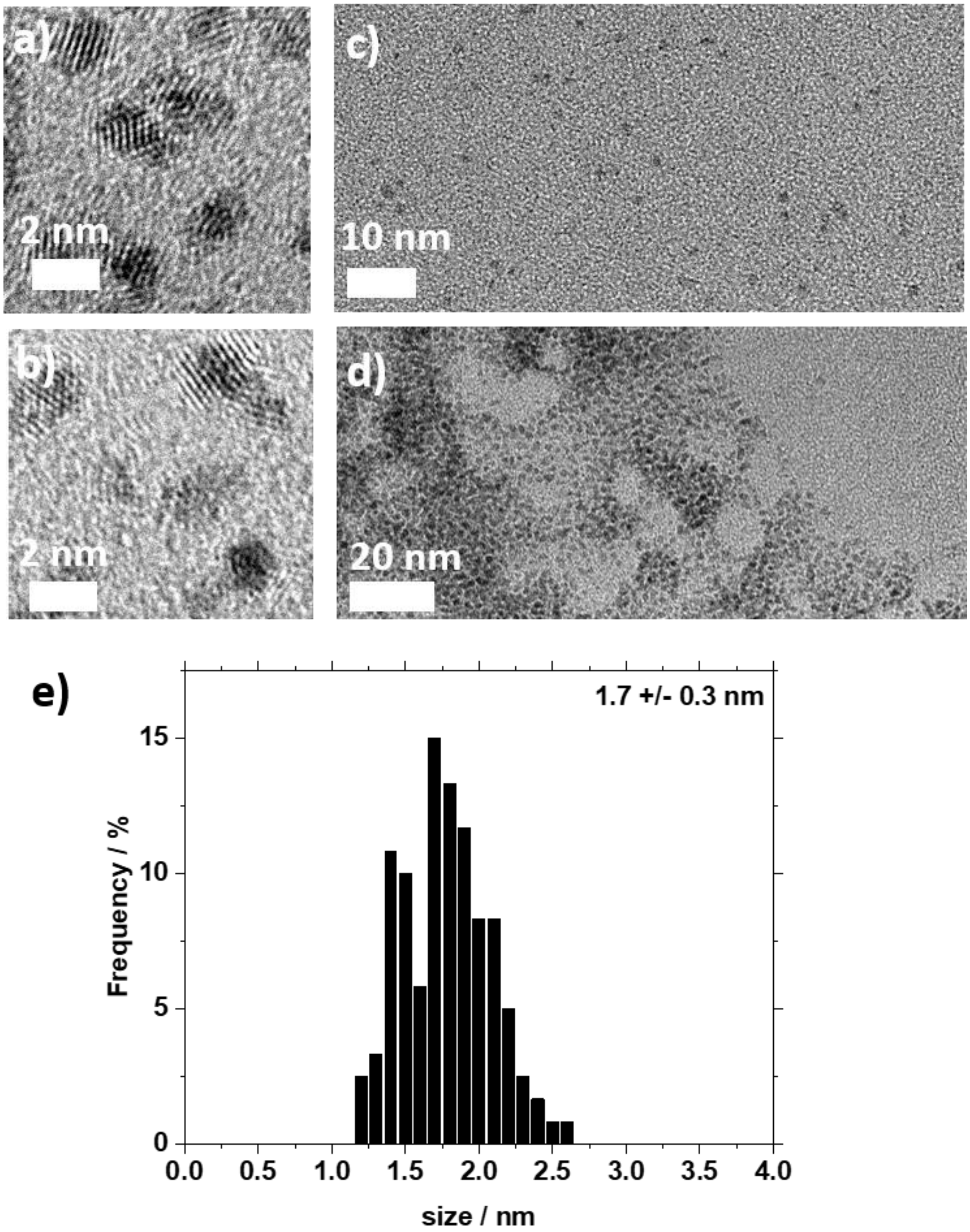

Figure S4 a-d) Illustrative TEM micrographs of Ir nanoparticles obtained by the mono-alcohol synthesis process at different magnifications. (e) Related size distribution.

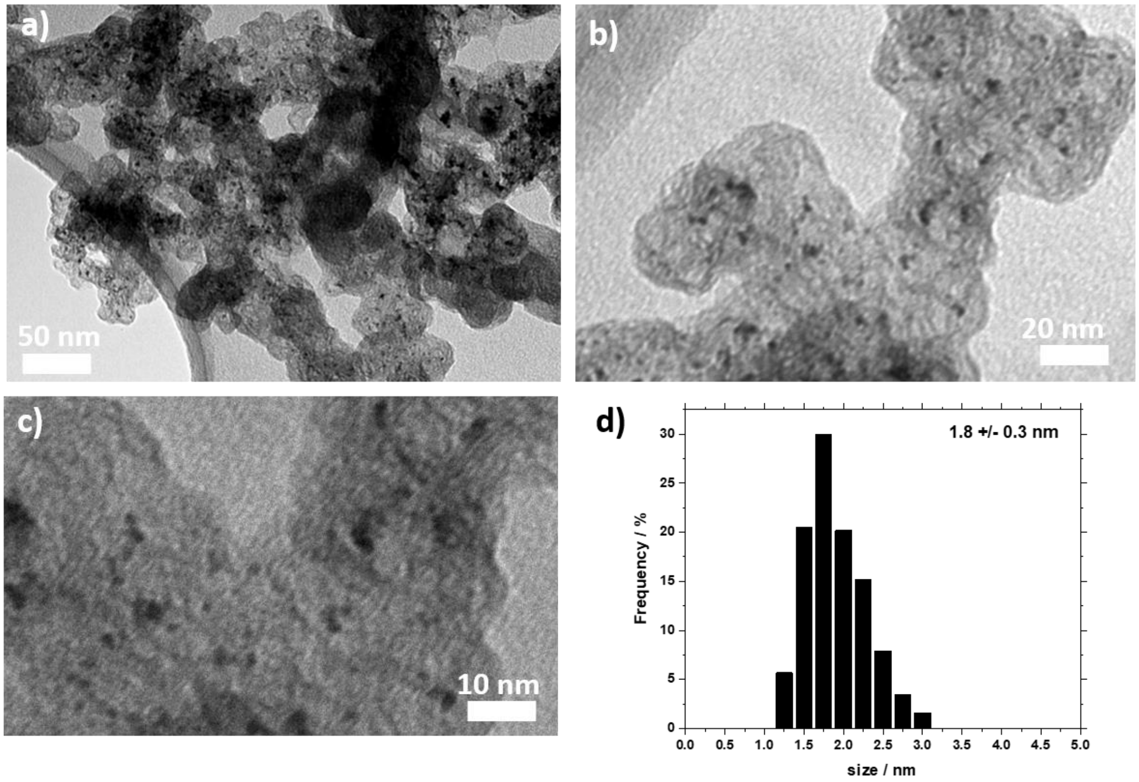

Figure S5 a-d) Illustrative TEM micrographs of the Ir nanoparticles distributed on carbon support at different magnifications. (e) Related size distribution.

Cyclic voltammograms

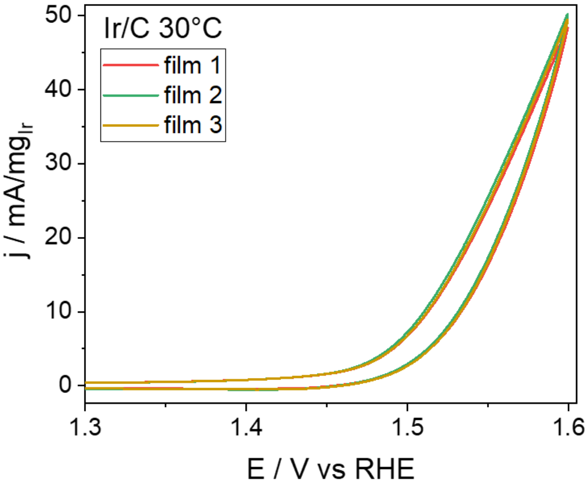

Figure S6 Cyclic voltammograms of Ir/C (50 wt%) deposited on MPL/GDL. The cyclic voltammograms of three different catalyst films with a nominal loading of 200 $\mu g_{Ir}/cm^2$ are shown

to demonstrate reproducibility. They were recorded at 30 degrees C with a scan rate of 10 mV/s in 4 M $HClO_4$ catalyst and applied iR correction

***Operando* electroreduction of surface oxide**

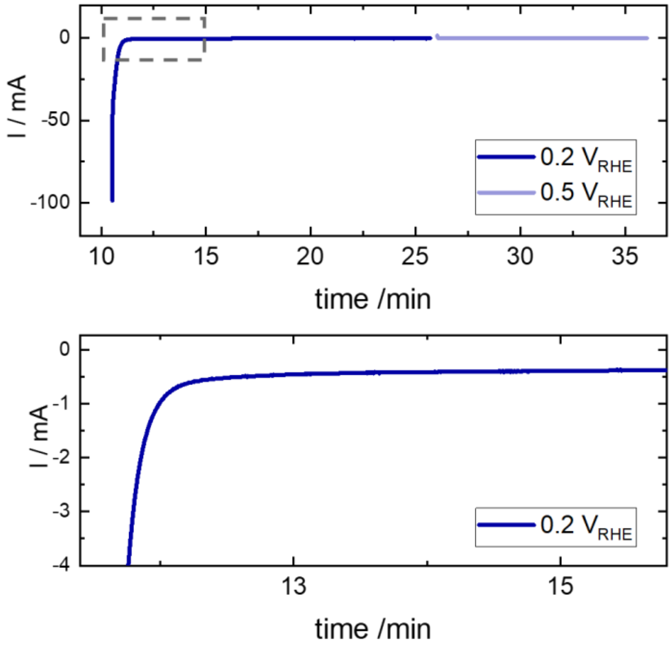

Figure S7 Currents collected as a function of time, while a reducing potential was applied in the electrochemical cell (0.2 and 0.5 V vs RHE), indicating the reduction of the surface oxide formed. The lower panel of the figure represents a zoom of the region indicated by the grey box. In the first minutes of potential hold at 0.2 V vs RHE a continuous reductive current was recorded.

Oxygen evolution

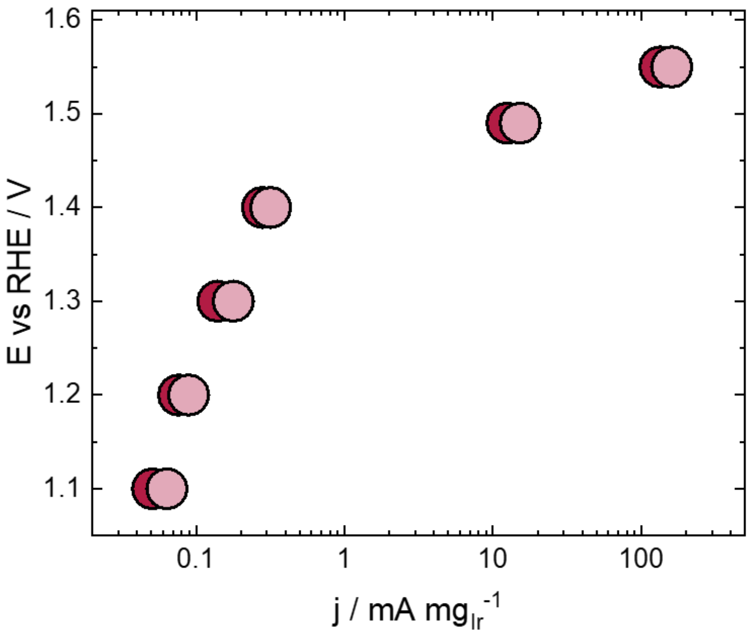

Figure S8 Ir-mass-based currents derived from the steady state measurements showing the recorded current densities (average of last 100 seconds) after applying the potential for 10 minutes. Two repeats are shown: Sample 1 is marked in dark red and Sample 2 (repeat) is represented in the lighter markers. The shown data have been post-corrected for residual uncompensated resistance after 85% online compensation. We note here that part of the current measured could also stem from corrosion of the carbon support.

Reproducibility of the measurements

Total scattering in reciprocal space

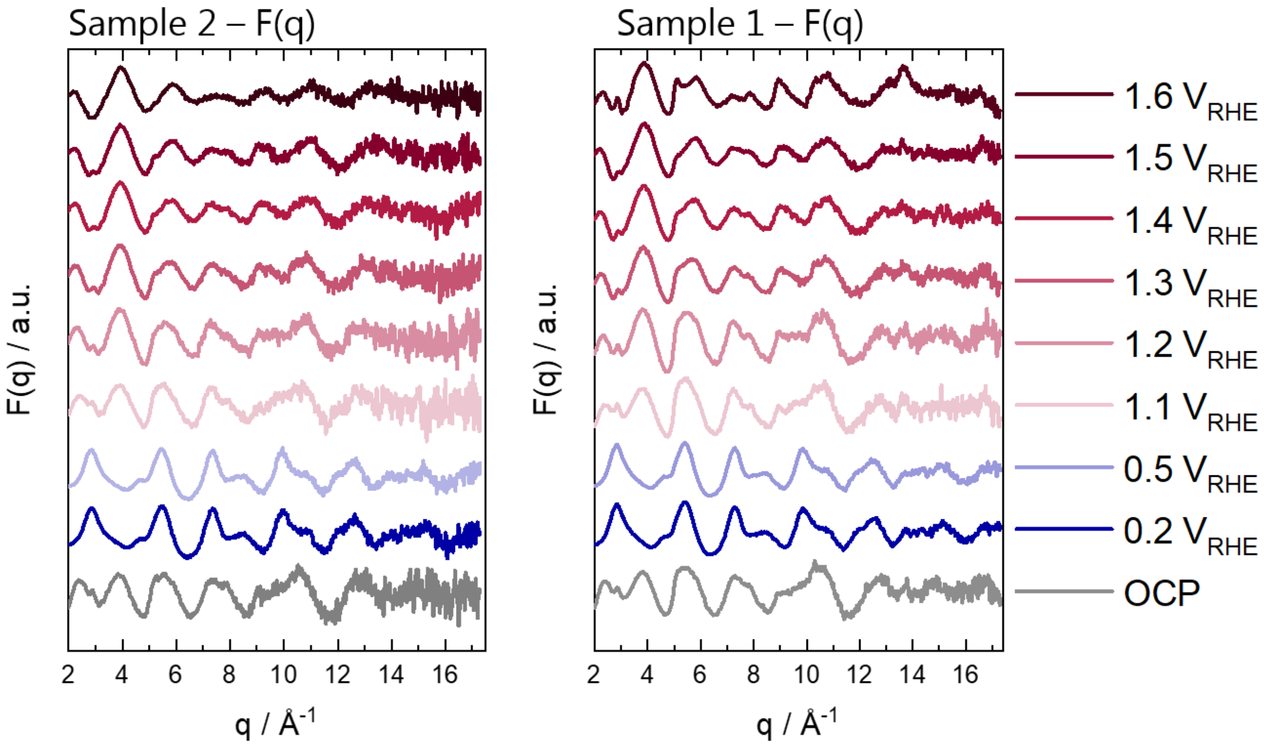

Figure S9 Experimental *operando* X-ray total scattering data of replicated experiments in reciprocal space *F(q).* The appearance of a small peak at 5.3 Å^{-1} at the highest potential in Sample 1 may be related to incomplete subtraction of the cell material, as evident in the not-background subtracted scattering data shown in **Figure S11,** compared to the PEEK reference.

Total scattering in real space

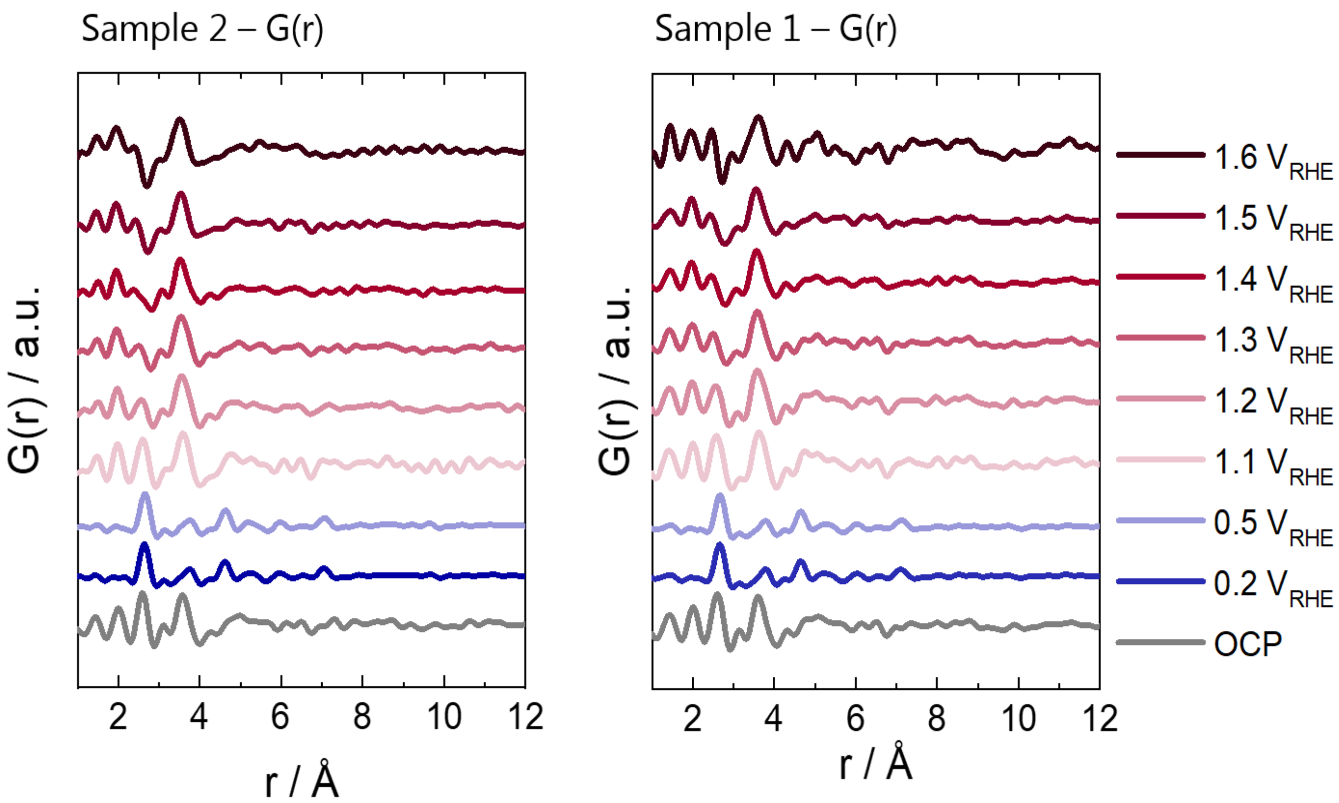

Figure S10 Experimental *operando* X-ray total scattering data of replicated experiments in real space *G(r).*

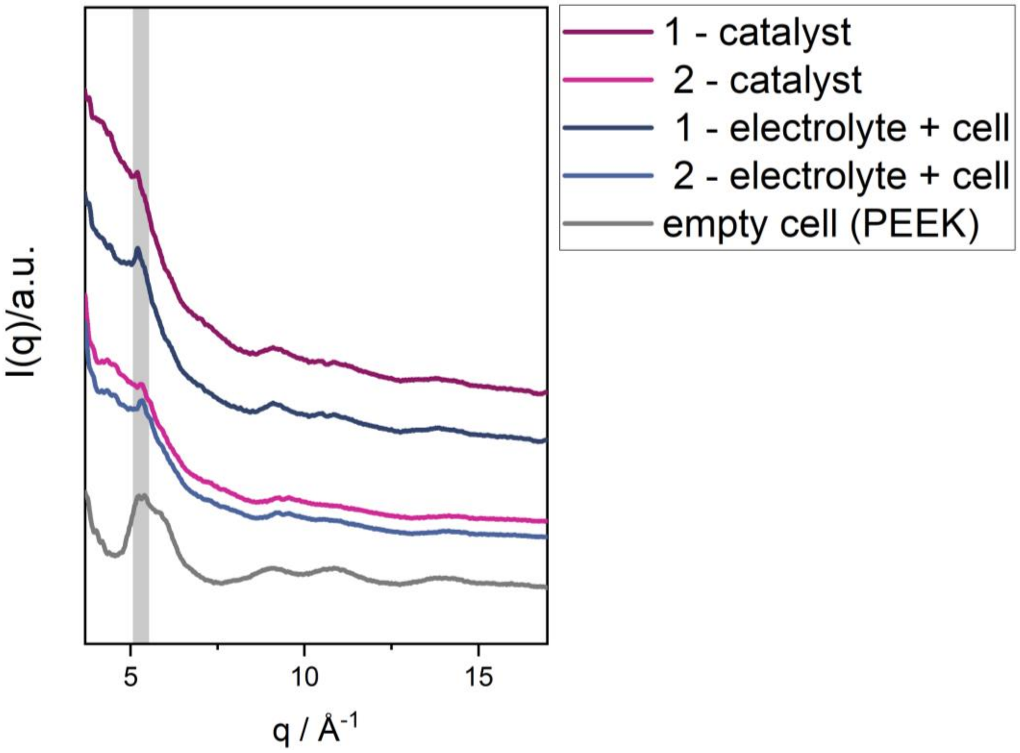

Figure S11 X-ray total scattering data collected at 1.6 V vs RHE of samples 1 and 2 from both the catalyst layer as well as the electrolyte, plus the X-ray total scattering signal of the empty cell. We show a zoom-in on the area from which the additional features evident in **Figures S9** and **S12** stems.

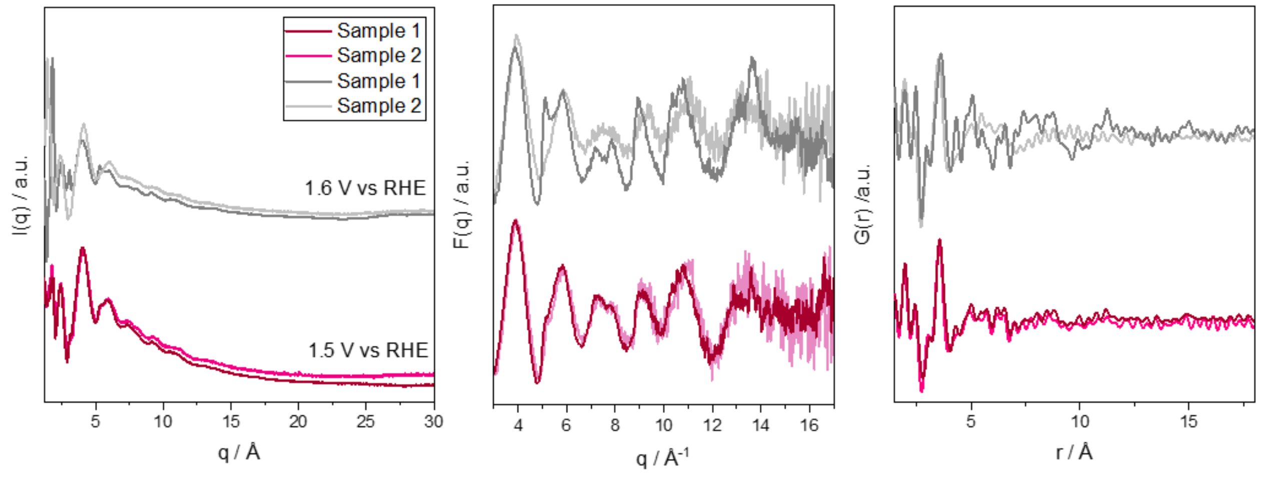

Figure S12 I(q), F(q), and G(r) data collected at 1.5 V and 1.6 V vs RHE for sample 1 and the repeat measurement, sample 2.

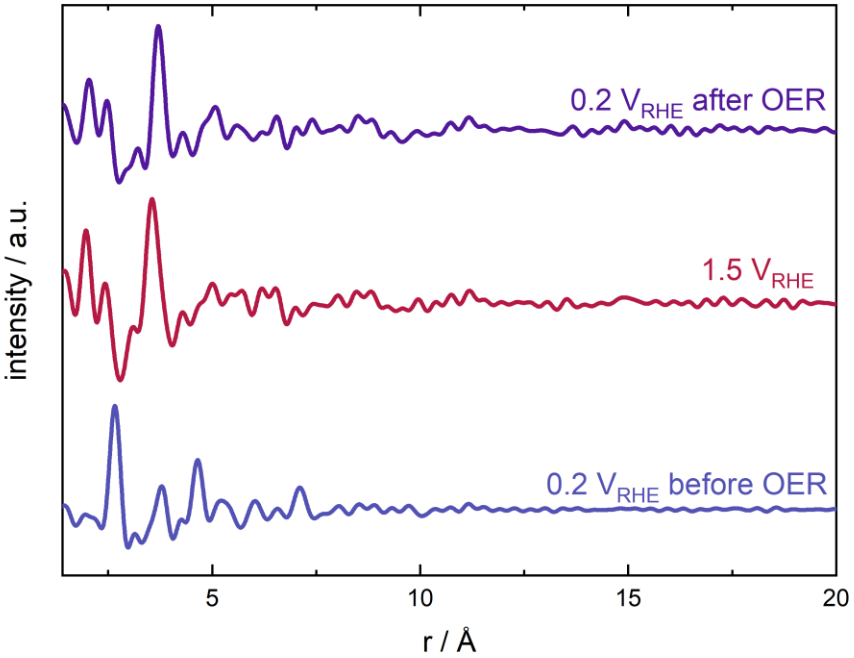

Figure S13 Experimental *operando* X-ray total scattering real space *G(r)* collected at 0.2 V vs RHE before electrochemical activation and OER (metallic), at 1.5 V vs RHE during OER (oxide) and collected again at 0.2 V vs RHE after OER, where the catalyst remains an oxide. This confirms the irreversibility of the activation process.

Structure of metallic nanoparticles after electroreduction

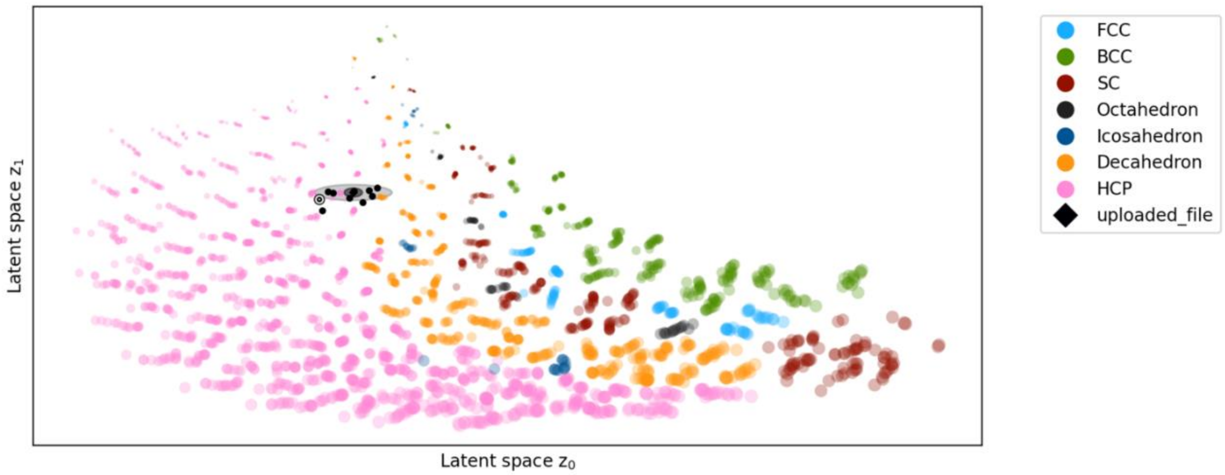

Figure S14 A two-dimensional latent space constructed by the deep learning algorithm DeepStruc[14], which is trained on PDFs from mono-metallic nanoparticles up to 200 atoms of seven different structure types: *fcc* (light blue), *bcc* (green), *sc* (red), octahedral (dark grey), icosahedral (dark blue), decahedral (orange), and *hcp* (pink). Each point in the latent space corresponds to a mono-metallic structure of up to 200 atoms based on its simulated PDF. Test data points are plotted on top of the training and validation data, which is made semi-transparent. The size of the points relates to the size of the embedded structure. The latent space locations of the reconstructed structures from our experimental PDF are shown with black markers. The test data PDF was obtained from Fourier transformation of the total scattering data using Q_{min} = 0.7 Å^{-1} and Q_{max} = 18 Å^{-1}.

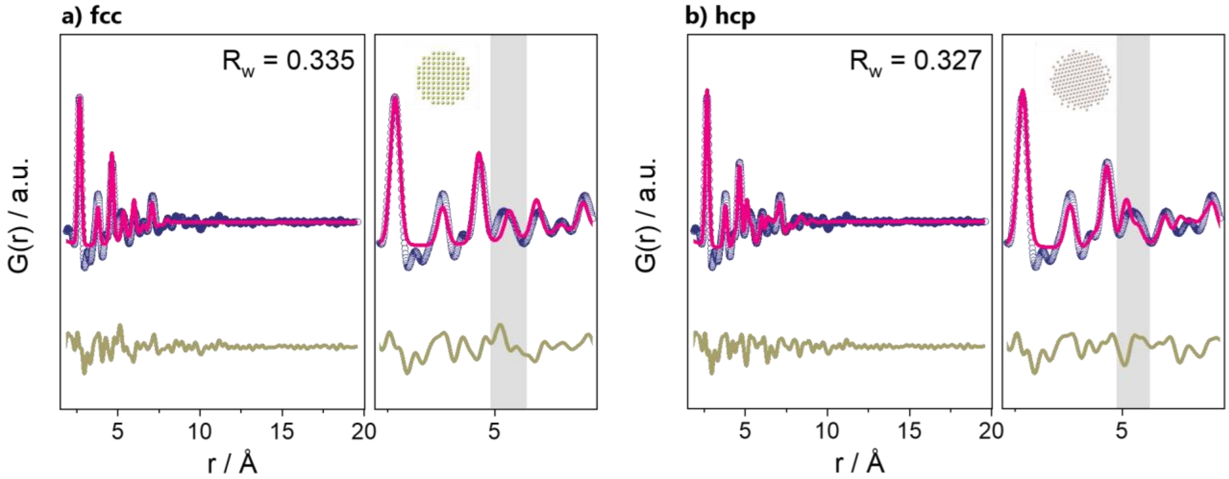

	fcc			*hcp*				*fcc/hcp ratio*	**R$_w$**
	***a* /Å**	**U_{iso} /Å^2**	**Sp /Å**	***a* /Å**	***c* /Å**	**U_{iso} /Å^2**	**Sp /Å**		
fcc only	3.79(3)	0.006(3)	9(2)						0.337
hcp only				2.69(3)	4.33(9)	0.005(2)	11(3)		0.329
two phase	3.82(6)	0.006(4)	13(8)	2.66(10)	4.32(17)	0.005(4)	10(5)	65wt% (29) /35wt% (29)	0.282

Figure S15 Plot of a PDF refinement using a) a single *fcc* phase and b) a single *hcp* phase, as well as refined values from PDF refinement using an *fcc*, or *hcp* phase, or both to describe the *operando* PDF of the electrochemically reduced metal.

Decahedral clusters

We validated our modeling approach using a brute-force structure-mining algorithm[8] that refines *fcc*, *hcp*, body-centered cubic (*bcc)*, and simple cubic (*sc)* structured clusters as well as octahedral, decahedral, and icosahedral clusters to the experimental PDF. This approach is limited to single phases. It identifies six structural models of decahedral particles with atom numbers between 100 and 200 atoms ($R_w \leq 0.35$) as the best-fitting structures. The decahedral clusters and corresponding fits are shown in SI, **Table S2,** and **Figure S16**. The fits were obtained following the modeling procedure described in Section 1 – Materials and Methods of the SI.

Table S2 All structures from the brute-force structure mining algorithm that was fitted to the experimental PDF with an R_w lower than 0.35. The decahedral clusters were constructed by ASE using the decahedral cluster module, defining each cluster through the parameters p (number of atoms on the (100) facets perpendicular to the five-fold axis), q (number of atoms on the (100) facets parallel to the five-fold axis, with $q = 1$ corresponding to no visible (100) facets, and r (depth of the m of re-entrance at the pentagon corners), as given in the table. The refined parameters from the DiffPy-CMI models are given for each cluster.

**p,q,r**	**3,1,1**	**2,1,2**	**3,3,1**	**4,1,1**	**2,3,1**	**2,4,1**
**R_w**	0.336	0.340	0.348	0.345	0.354	0.353
**Atoms**	100	156	192	176	101	127
**p**	3	2	3	4	2	2
**q**	1	1	3	1	3	4
**r**	1	2	1	1	1	1
**Front view** 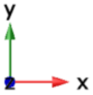	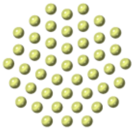	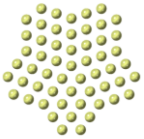	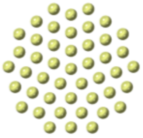	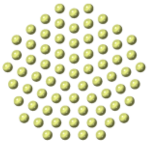	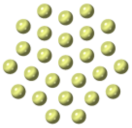	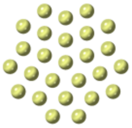
**d/nm**	1.6	1.9	1.6	2.0	1.2	1.2
**Side view** 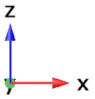	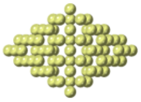	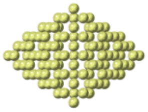	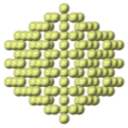	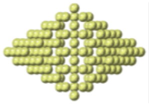	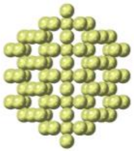	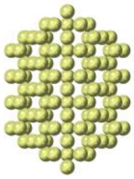
**d/nm**	1.1	1.3	1.6	1.3	1.3	1.6

DiffPy-CMI						
**R_w**	**0.333**	**0.327**	**0.314**	**0.330**	**0.344**	**0.340**
**U_{iso} / Å^2**	0.001(1)	0.001(1)	0.001(1)	0.001(1)	0.001(1)	0.001(1)
**δ_2 / Å^2**	3(5)	4(3)	4(3)	4(3)	4(4)	4(4)
**scale**	0.09(2)	0.09(2)	0.08(2)	0.08(2)	0.09(2)	0.09(2)
**Zoom scale r**	0.993(6)	0.993(6)	0.993(6)	0.993(6)	0.993(7)	0.994(7)

p,q,r = 3,1,1

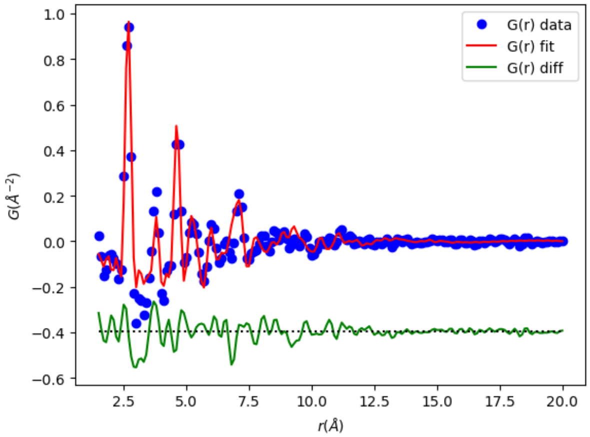

p,q,r = 2,1,2

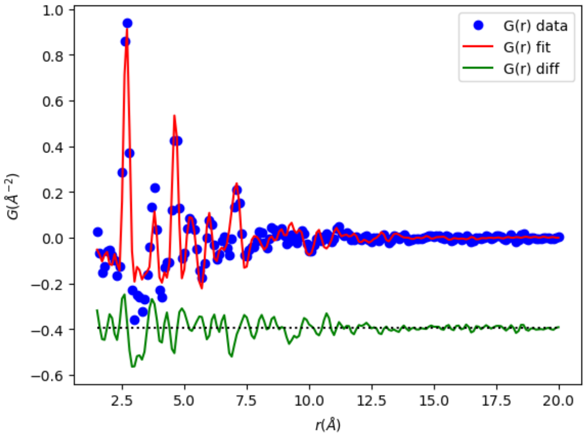

p,q,r = 3,3,1

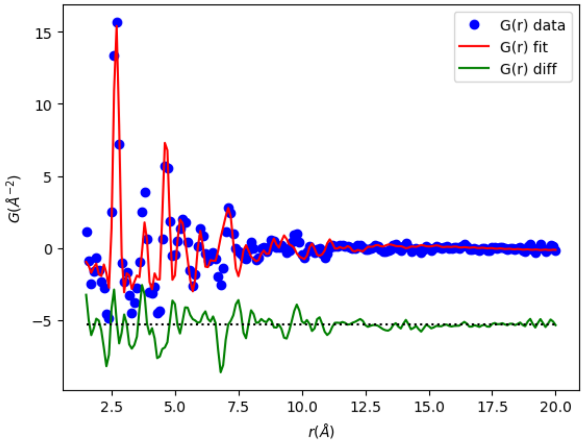

p,q,r = 4,1,1

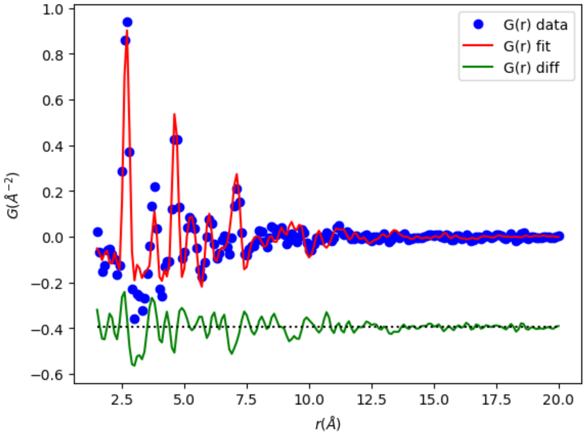

p,q,r = 2,3,1

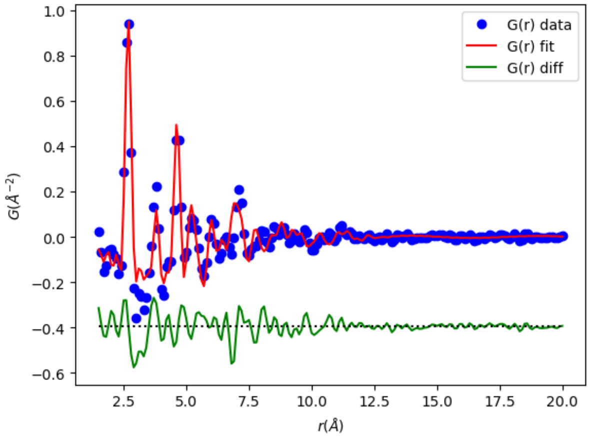

p,q,r = 2,4,1

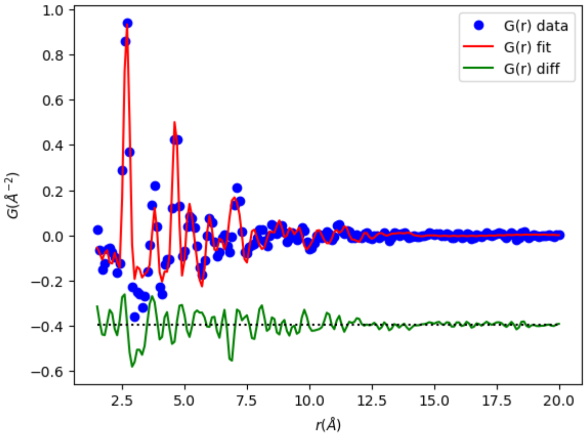

Figure S16. Plots of the PDF refinements corresponding to the decahedral models which are presented in **Table S2**. The models are identified by their p,q,r values which are used to construct the decahedral clusters.

Structure of electrochemically formed iridium oxide

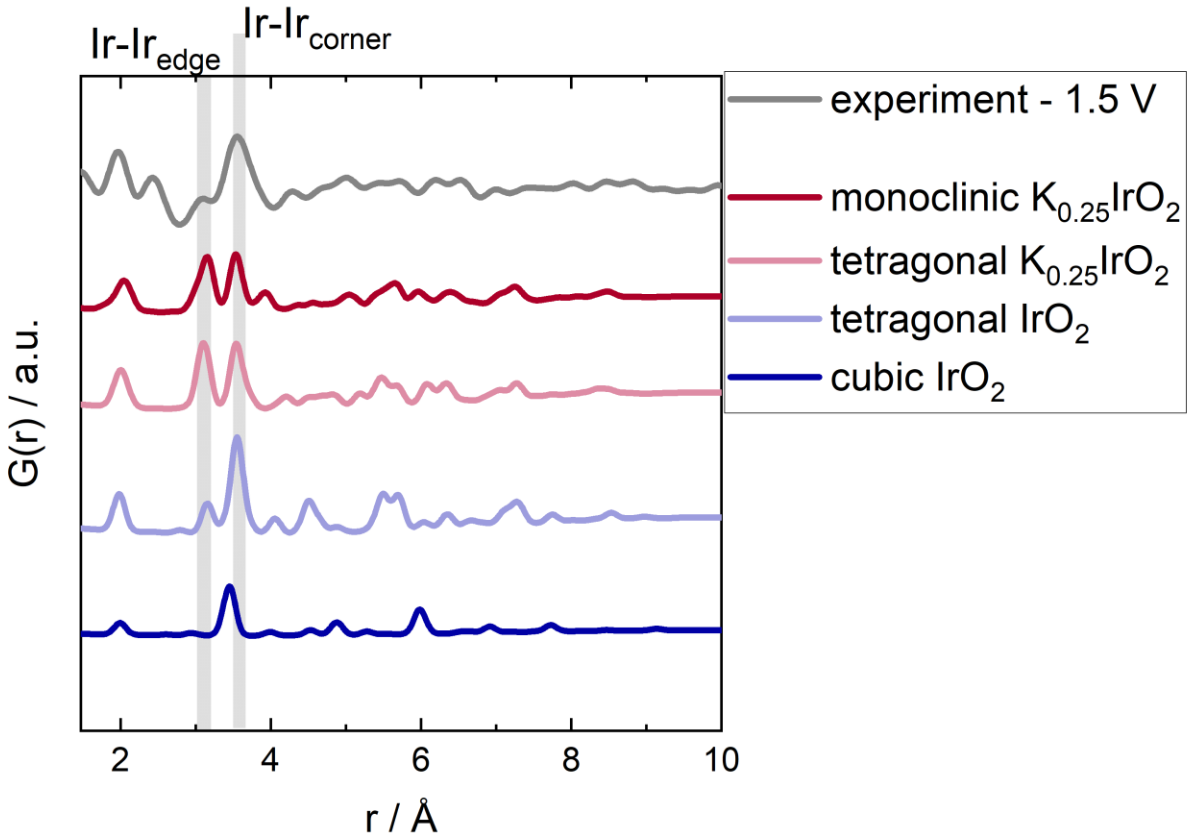

Figure S17 Calculated PDFs of different iridium oxide structures $U_{iso} = 0.003 Å^2$, $Spdiameter = 10$ Å), the hollandite structures both have a potassium occupation of 0.25 to largely maintain the iridium oxidation state compared to the experimental PDF obtained for the sample at 1.5 V vs RHE.

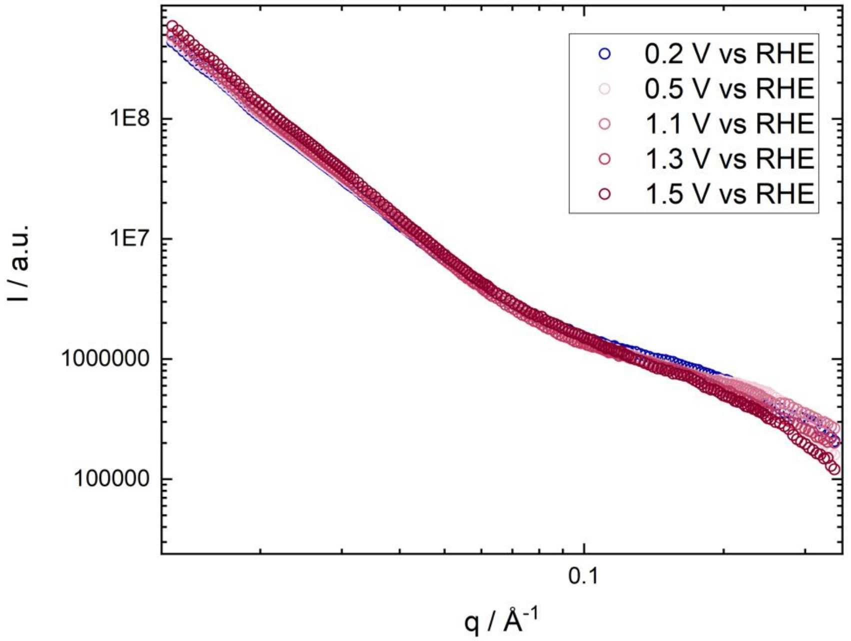

Figure S18 Overview of SAXS curves collected at different applied potentials in the *operando* cell.

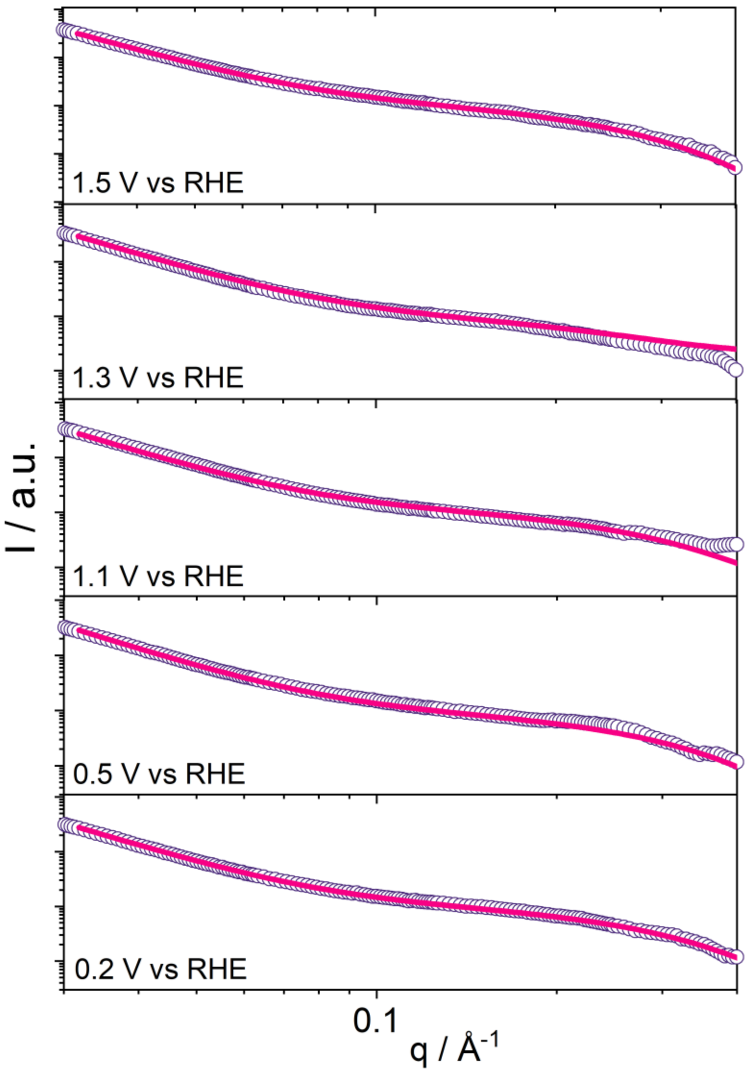

Figure S19 Fit of polydisperse spheres to the SAXS data collected at the different applied potentials. The fit variables are listed in Table S1.

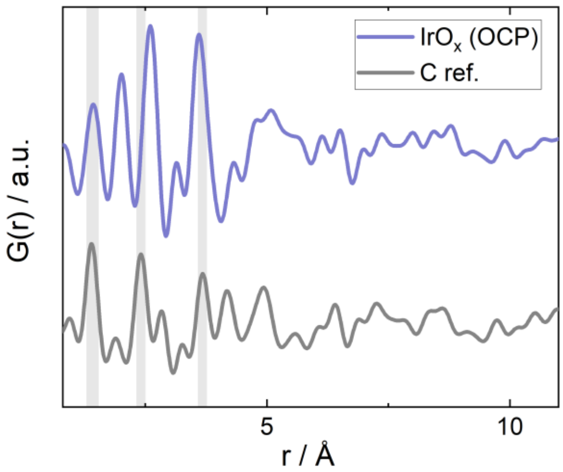

Figure S20 Experimental Carbon reference PDF, shown in light grey, collected *ex situ* of a gas diffusion electrode with porous carbon support deposited on top, sandwiched between Kapton tapes for measurement. The *operando* iridium oxide PDF with carbon contribution collected at open circuit potential is shown in light blue for comparison.

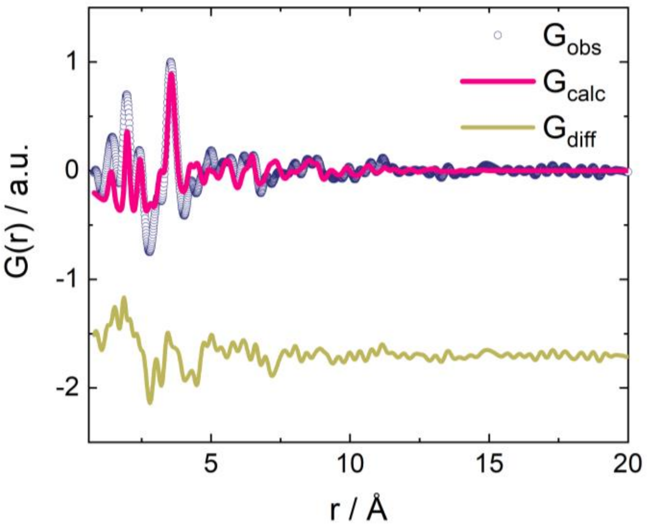

**Potenial vs RHE**	**R_w**	**Sp diameter / Å**	***a* / Å**	***c* /Å**	**U_{iso} (Ir) /Å^2**	**U_{iso} (O) / Å^2**	**δ_2 (Å^2)**	**Scale IrO_2**	**Scale C**
**1.3 V**	0.64	9(2)	4.58(6)	3.14(8)	0.009(6)	0.02(9)	3(2)	0.18(6)	0.04(2)
**1.4 V**	0.67	8(2)	4.52(5)	3.15(7)	0.006(5)	0.01(4)	3(2)	0.15(5)	0.04(2)
**1.5 V**	0.66	8(2)	4.51(5)	3.19(9)	0.010(6)	0.05(11)	4(1)	0.19(6)	0.03(2)
**1.6 V**	0.63	9(2)	4.56(6)	3.12(9)	0.017(8)	0.11(6)	4(2)	0.24(7)	0.06(2)

Figure S21 Plot of a representative PDF refinement (1.5 V vs RHE) and refined values from two-phase PDF refinement using a model of both rutile structure and a graphite phase to describe the *operando* PDFs of the electrochemically formed oxide phase.

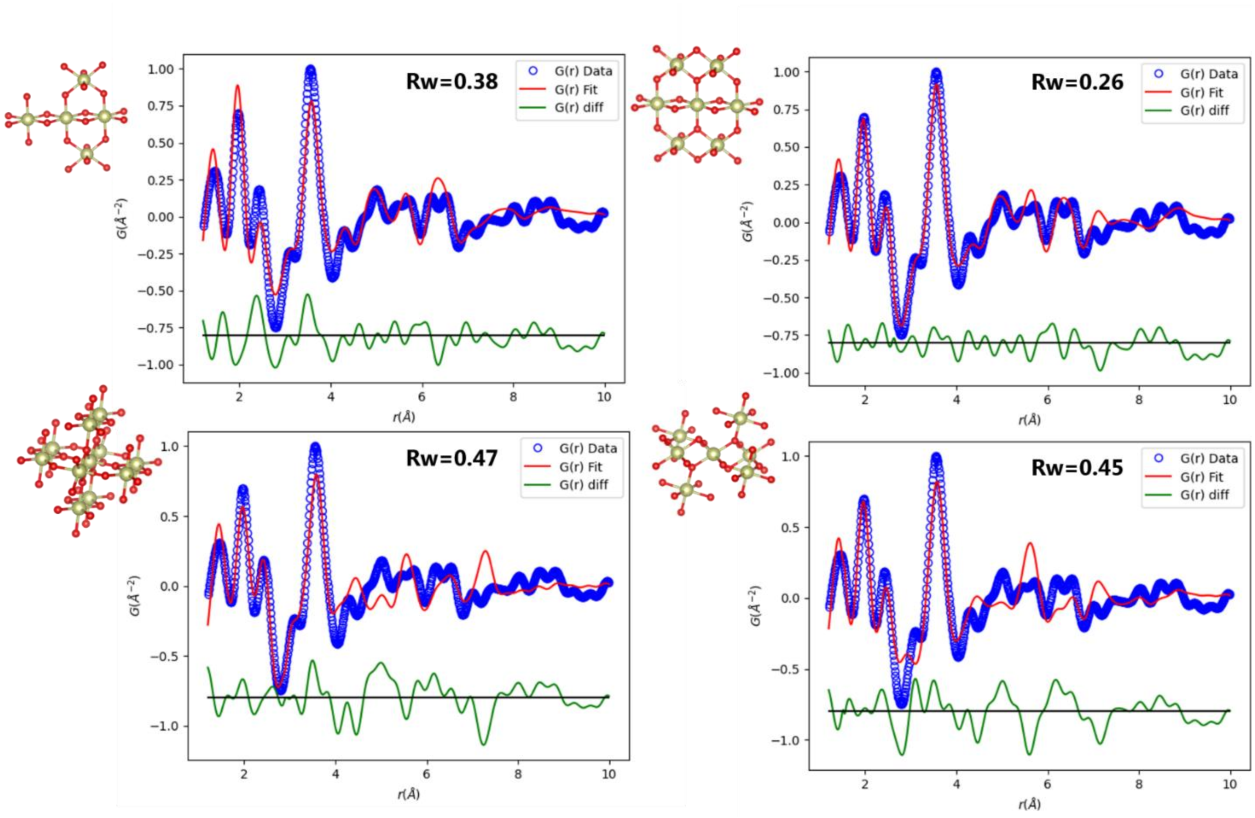

Figure S22: Fit of different cluster models cut out from the crystalline IrO_2 rutile structure to the PDF data collected at 1.5 V vs RHE showing the goodness factor of the fit.

Table S3 Refined values from cluster PDF refinements using a model of a rutile-structured IrOx cluster and a single sheet graphite carbon phase to describe the *operando* PDFs of the electrochemically formed oxide phase, and ball and stick model to show the atomic structure of the iridium oxide cluster used in the model.

	**Rw**	**U_{iso} Ir / Å^2**	**U_{iso} O / Å^2**	**U_{iso} C / Å^2**	**Ir-O_1 /Å CN=2**	**Ir-O_2 /Å CN=4**	**Ir-Ir_{edge} /Å**	**Ir-Ir_{corner} /Å**	**Scale IrO_2**	**Scale C**	**Zoom scale cluster**
**1.1 V**	0.57	0.011 (5)	0.006 (3)	0.020 (1)	1.97(1)	2.01(1)	3.17(2)	3.57(2)	0.37(1)	0.45(2)	1.005 (6)
**1.2 V**	0.42	0.011 (4)	0.010 (3)	0.014 (6)	1.95(1)	1.99(1)	3.15(2)	3.54(2)	0.40(3)	0.33(4)	0.996 (6)
**1.3 V**	0.33	0.011 (5)	0.013 (1)	0.010 (5)	1.95(2)	1.99(2)	3.14(3)	3.54(3)	0.37(6)	0.25(5)	0.995 (8)
**1.4 V**	0.28	0.007 (3)	0.013 (1)	0.011 (4)	1.95(1)	1.98(1)	3.14(2)	3.53(2)	0.36(5)	0.24(5)	0.993 (7)
**1.5 V**	0.26	0.011 (3)	0.013 (1)	0.013 (4)	1.94(1)	1.98(1)	3.13(2)	3.52(3)	0.38(5)	0.25(5)	0.989 (7)
**1.6 V**	0.33	0.014 (6)	0.013 (1)	0.004 (1)	1.94(1)	1.98(1)	3.13(2)	3.52(3)	0.41(4)	0.23(3)	0.990 (7)

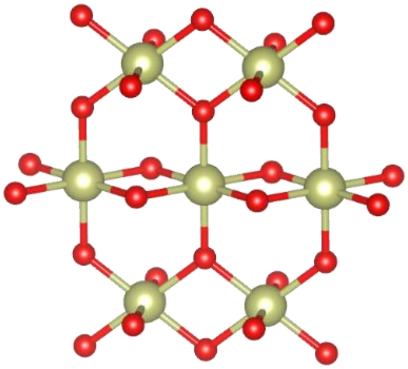

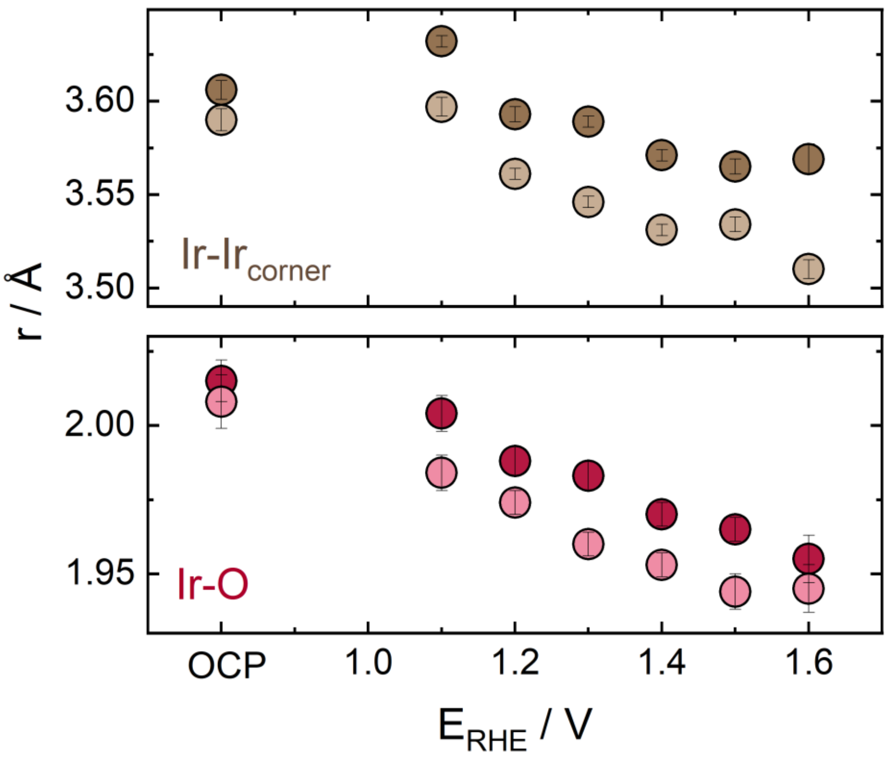

Figure S23 Evolution of Ir-Ir distance of corner-sharing octahedra and Ir-O distances, extracted from the position of the peaks in the PDFs (determined from Gaussian fits of the peaks) as a function of applied potential. A repeated measurement is added (light markers) to confirm the general trends

Operando XAS analysis

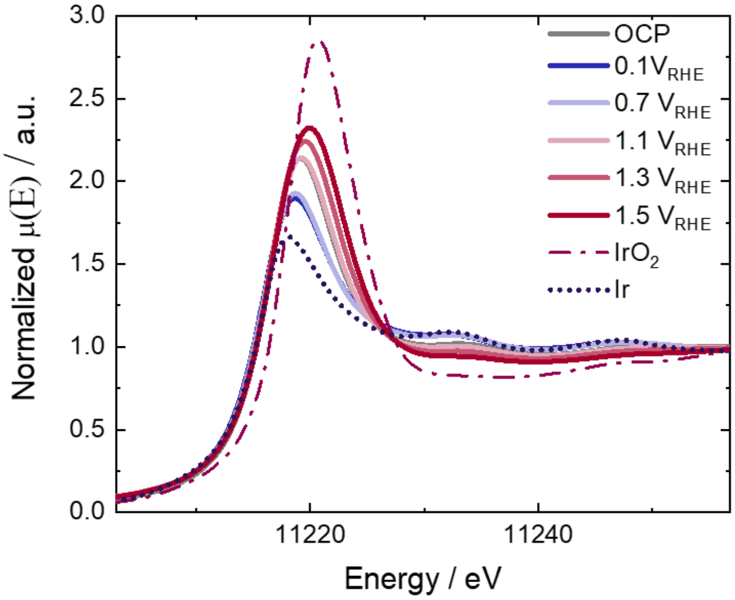

Figure S24 *Operando* XANES data collected at the Iridium L_3 edge of the iridium catalyst including spectra of a metallic iridium reference foil and an IrO_2 powder pellet reference.

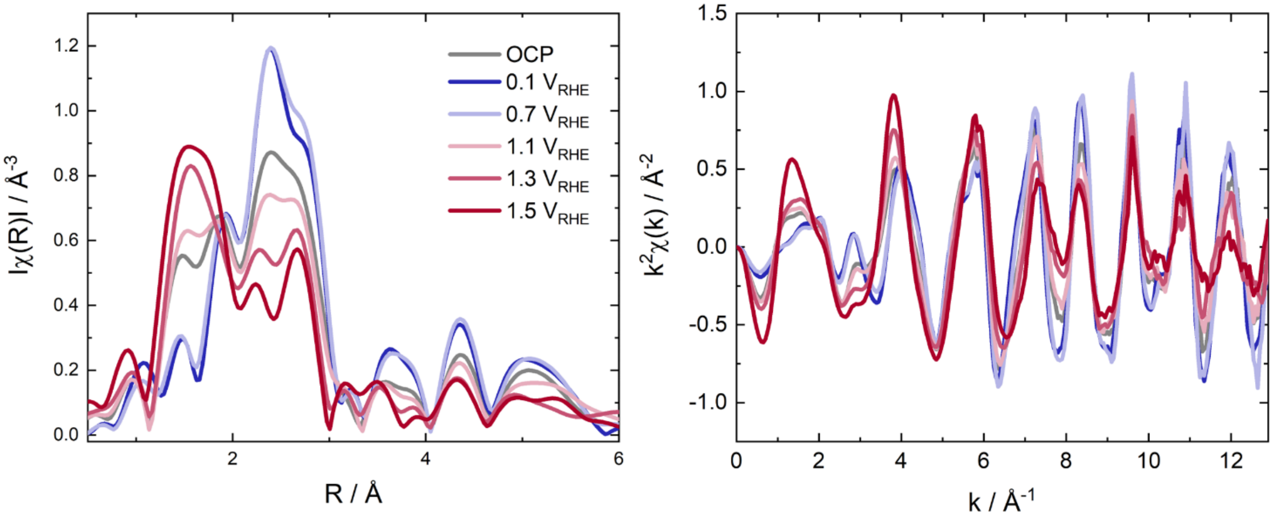

Figure S25 R-space representation without applied phase shift left, and k-space representation (k^2 weighted), right, of the *operando* EXAFS data collected at a row of potentials going from OCP first to reducing conditions and further to oxidizing potentials, where oxygen evolution is observed.

Table S4 Results of the EXAFS modeling using a two-shell model containing iridium oxide and metallic iridium contributions to describe the *operando* data, as well as from the linear combination fitting of the XANES data using the references collected for metallic Iridium and IrO_2 powder as the respective components.

**Potential**	**Ex situ**	**0.1 V vs RHE**	**0.7 Vs RHE**	**1.1V vs RHE**	**1.3 V vs RHE**	**1.5 V vs RHE**
**E_0 / eV**	11223(1)	11222(1)	11223(1)	11223(2)	11223(2)	11223(3)
**Metal ratio**	0.68+/-0.05	1	0.82+/-0.09	0.67+/-0.1	0.56+/-0.1	0.37+/- 0.1
**Ir-Ir / Å**	2.69(1)	2.69(1)	2.69(1)	2.69(2)	2.69(1)	2.70(1)
**Oxide ratio**	0.32+/-0.04	-	0.18+/-0.06	0.33+/-0.1	0.44+/-0.1	0.63 +/- 0.1
**Ir-O_1 / Å (N=4)**	1.97(1)	-	1.98(2)	1.97(1)	1.95(2)	1.95(2)
**Ir-O_2 / Å (N=2)**	1.97(1)	-	1.98(2)	1.97(1)	1.95(2)	1.93(1)
**Ir-Ir / Å**	3.1(1)		3.2(1)	3.1(1)	3.0(1)	3.0(1)
**DW_{Ir} / $Å^2$**	0.005(1)	0.005(1)	0.005(1)	0.006(1)	0.006(1)	0.009(3)
**DW_O / $Å^2$**	0.005(2)	-	0.005(1)	0.005(3)	0.005(1)	0.008(4)
**R-Factor**	0.010	0.010	0.005	0.013	0.018	0.031
**XANES LCA**	**Ex situ**	**0.1 V vs RHE**	**0.7 Vs RHE**	**1.1V vs RHE**	**1.3 V vs RHE**	**1.5 V vs RHE**
**Metal ratio**	0.616(4)	0.982(6)	0.793(5)	0.597(4)	0.477(4)	0.373(1)
**Oxide ratio**	0.384(4)	0.018(7)	0.207(7)	0.403(4)	0.523(4)	0.672(4)
**R-factor**	0.002	0.003	0.003	0.002	0.001	0.001

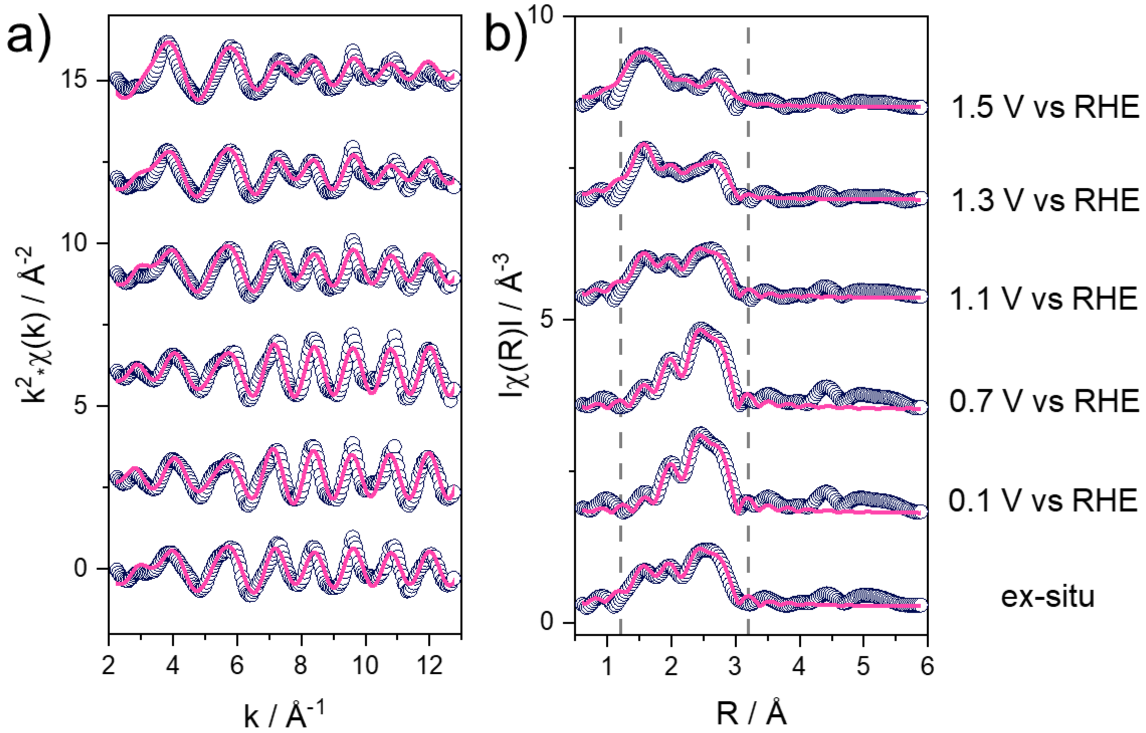

Figure S26 Experimental data (o) and least squares refined (–) fits corresponding to Table S4 of a) the $k^2\chi$ (k) and (b) the EXAFS Fourier transforms (k^2 weighted, k-range = 3-13 $Å^{-1}$) of all spectra recorded at the Ir L_3 edge. The fitting range of the FT-EXAFS (R =1.2-3.2 Å) is highlighted with the grey dashed lines in b).

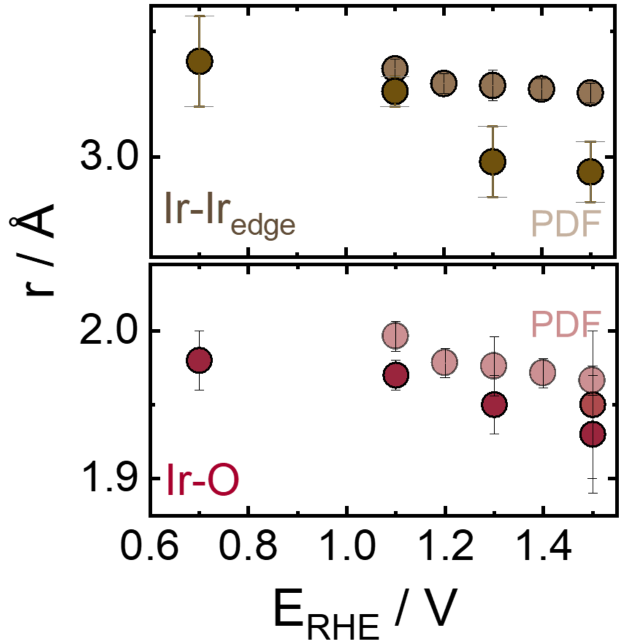

Figure S27 Distances extracted from modeling the *operando* EXAFS data for the Ir-O bond and Ir-Ir edge distance in iridium oxide in comparison to the *operando* PDF fits, from Figure 4 shown in lighter colors.

References

(1) Bornet, A.; Pittkowski, R.; Nielsen, T. M.; Berner, E.; Maletzko, A.; Schröder, J.; Quinson, J.; Melke, J.; Jensen, K. M. Ø.; Arenz, M. Influence of Temperature on the Performance of Carbon- and ATO-Supported Oxygen Evolution Reaction Catalysts in a Gas Diffusion Electrode Setup. *ACS Catal.* **2023**, 7568–7577. https://doi.org/10.1021/acscatal.3c01193.

(2) Inaba, M.; Quinson, J.; Arenz, M. pH Matters: The Influence of the Catalyst Ink on the Oxygen Reduction Activity Determined in Thin Film Rotating Disk Electrode Measurements. *J. Power Sources* **2017**, *353*, 19–27. https://doi.org/10.1016/j.jpowsour.2017.03.140.

(3) Martens, I.; Chattot, R.; Rasola, M.; Blanco, M. V.; Honkimäki, V.; Bizzotto, D.; Wilkinson, D. P.; Drnec, J. Probing the Dynamics of Platinum Surface Oxides in Fuel Cell Catalyst Layers Using in Situ X-Ray Diffraction. *ACS Appl. Energy Mater.* **2019**, *2* (11), 7772–7780. https://doi.org/10.1021/acsaem.9b00982.

(4) Kieffer, J.; Karkoulis, D. PyFAI, a Versatile Library for Azimuthal Regrouping. *J. Phys. Conf. Ser.* **2013**, *425* (20), 202012. https://doi.org/10.1088/1742-6596/425/20/202012.

(5) Schröder, J.; Pittkowski, R. K.; Martens, I.; Chattot, R.; Drnec, J.; Quinson, J.; Kirkensgaard, J. J. K.; Arenz, M. Tracking the Catalyst Layer Depth-Dependent Electrochemical Degradation of a Bimodal Pt/C Fuel Cell Catalyst: A Combined Operando Small- and Wide-Angle X-Ray Scattering Study. *ACS Catal.* **2022**, *12* (3), 2077–2085. https://doi.org/10.1021/acscatal.1c04365.

(6) Juhás, P.; Davis, T.; Farrow, C. L.; Billinge, S. J. L. PDFgetX3: A Rapid and Highly Automatable Program for Processing Powder Diffraction Data into Total Scattering Pair Distribution Functions. *J. Appl. Crystallogr.* **2013**, *46* (2), 560–566. https://doi.org/10.1107/S0021889813005190.

(7) Briois, V.; Fontaine, C. L.; Belin, S.; Barthe, L.; Moreno, T.; Pinty, V.; Carcy, A.; Girardot, R.; Fonda, E. ROCK: The New Quick-EXAFS Beamline at SOLEIL. J. Phys. Conf. Ser. 2016, 712 (1), 012149. https://doi.org/10.1088/1742-6596/712/1/012149.

(8) La Fontaine, C.; Belin, S.; Barthe, L.; Roudenko, O.; Briois, V. ROCK: A Beamline Tailored for Catalysis and Energy-Related Materials from Ms Time Resolution to Mm Spatial Resolution. Synchrotron Radiat. News 2020, 33 (1), 20–25. https://doi.org/10.1080/08940886.2020.1701372.

(9) Ravel, B.; Newville, M. ATHENA, ARTEMIS, HEPHAESTUS: Data Analysis for X-Ray Absorption Spectroscopy Using IFEFFIT. *J. Synchrotron Radiat.* **2005**, 12 (4), 537–541. https://doi.org/10.1107/S0909049505012719.

(10) Hamar Reksten, A.; E. Russell, A.; W. Richardson, P.; J. Thompson, S.; Mathisen, K.; Seland, F.; Sunde, S. Strategies for the Analysis of the Elemental Metal Fraction of Ir and Ru Oxides via XRD, XANES, and EXAFS. *Phys. Chem. Chem. Phys.* **2019**, *21* (23), 12217–12230. https://doi.org/10.1039/C9CP01758E.

(11) Alumina Supported Rhodium Catalyst. The Formation of a New Metal-Metal Oxide Interface Determined with EXAFS. *J. Phys. Chem.* **1989**, *93* (8), 3179–3185.

(12) Bizzotto, F.; Quinson, J.; Zana, A.; Kirkensgaard, J. J. K.; Dworzak, A.; Oezaslan, M.; Arenz, M. Ir Nanoparticles with Ultrahigh Dispersion as Oxygen Evolution Reaction (OER) Catalysts: Synthesis and Activity Benchmarking. *Catal. Sci. Technol*. **2019**, 9 (22), 6345–6356. https://doi.org/10.1039/C9CY01728C.

(13) Binninger, T.; Fabbri, E.; Patru, A.; Garganourakis, M.; Han, J.; Abbott, D. F.; Sereda, O.; Kötz, R.; Menzel, A.; Nachtegaal, M.; Schmidt, T. J. Electrochemical Flow-Cell Setup for

In Situ X-Ray Investigations: I. Cell for SAXS and XAS at Synchrotron Facilities. *J. Electrochem. Soc*. **2016**, 163 (10), H906–H912. https://doi.org/10.1149/2.0201610jes
Juhás, P.; Davis, T.; Farrow, C. L.; Billinge, S. J. L. PDFgetX3: A Rapid and Highly Automatable Program for Processing Powder Diffraction Data into Total Scattering Pair Distribution Functions. *J. Appl. Crystallogr.* **2013**, *46* (2), 560–566. https://doi.org/10.1107/S0021889813005190.
(14) S. Kjær, E. T.; S. Anker, A.; N. Weng, M.; L. Billinge, S. J.; Selvan, R.; Ø. Jensen, K. M. DeepStruc: Towards Structure Solution from Pair Distribution Function Data Using Deep Generative Models. *Digit. Discov.* **2023**, *2* (1), 69–80. https://doi.org/10.1039/D2DD00086E.
(15) Banerjee, S.; Liu, C.-H.; Jensen, K. M. Ø.; Juhás, P.; Lee, J. D.; Tofanelli, M.; Ackerson, C. J.; Murray, C. B.; Billinge, S. J. L. Cluster-Mining: An Approach for Determining Core Structures of Metallic Nanoparticles from Atomic Pair Distribution Function Data. *Acta Crystallogr. Sect. Found. Adv.* **2020**, *76* (1), 24–31. https://doi.org/10.1107/S2053273319013214.
(16) Larsen, A. H.; Mortensen, J. J.; Blomqvist, J.; Castelli, I. E.; Christensen, R.; Dułak, M.; Friis, J.; Groves, M. N.; Hammer, B.; Hargus, C.; Hermes, E. D.; Jennings, P. C.; Jensen, P. B.; Kermode, J.; Kitchin, J. R.; Kolsbjerg, E. L.; Kubal, J.; Kaasbjerg, K.; Lysgaard, S.; Maronsson, J. B.; Maxson, T.; Olsen, T.; Pastewka, L.; Peterson, A.; Rostgaard, C.; Schiøtz, J.; Schütt, O.; Strange, M.; Thygesen, K. S.; Vegge, T.; Vilhelmsen, L.; Walter, M.; Zeng, Z.; Jacobsen, K. W. The Atomic Simulation Environment—a Python Library for Working with Atoms. *J. Phys. Condens. Matter* **2017**, *29* (27), 273002. https://doi.org/10.1088/1361-648X/aa680e.
(17) Juhás, P.; Farrow, C.; Yang, X.; Knox, K.; Billinge, S. Complex Modeling: A Strategy and Software Program for Combining Multiple Information Sources to Solve Ill Posed Structure and Nanostructure Inverse Problems. *Acta Crystallogr. Sect. Found. Adv.* **2015**, *71* (6), 562–568. https://doi.org/10.1107/S2053273315014473.
(18) Proffen, T.; Billinge, S. J. L. PDFFIT, a Program for Full Profile Structural Refinement of the Atomic Pair Distribution Function. *J. Appl. Crystallogr.* **1999**, *32* (3), 572–575. https://doi.org/10.1107/S0021889899003532.
(19) Farrow, C. L.; Juhas, P.; Liu, J. W.; Bryndin, D.; Božin, E. S.; Bloch, J.; Proffen, T.; Billinge, S. J. L. PDFfit2 and PDFgui: Computer Programs for Studying Nanostructure in Crystals. *J. Phys. Condens. Matter* **2007**, *19* (33), 335219. https://doi.org/10.1088/0953-8984/19/33/335219.
(20) Zobel, M.; Neder, R. B.; Kimber, S. A. J. Universal Solvent Restructuring Induced by Colloidal Nanoparticles. *Science* **2015**, *347* (6219), 292–294. https://doi.org/10.1126/science.1261412.
(21) SasView. *SasView*. https://sasview.github.io/ (accessed 2023-07-20).
